# Stage‐Associated Microglial Subpopulations and Dynamics in Vascular Pathogenesis of Oxygen‐Induced Retinopathy

**DOI:** 10.1111/cpr.70165

**Published:** 2026-01-22

**Authors:** Yuan Ma, Ziye Chen, Baoyi Liu, Wen Ding, Runping Duan, Kangjie Kong, Zhuojun Xu, Jizhu Li, Jiali Ru, Dianlei Guo, Xiaoyue Wei, Yaping Liu, Zhuangling Lin, Yang Meng, Yuan Liu, Lan Jiang, Zitong Chen, Rebiya Tuxun, Chinling Tsai, Chunqiao Liu, Tao Li

**Affiliations:** ^1^ State Key Laboratory of Ophthalmology, Zhongshan Ophthalmic Center, Sun Yat‐sen University, Guangdong Provincial Key Laboratory of Ophthalmology and Visual Science Guangzhou China

**Keywords:** microglia, oxygen‐induced retinopathy, phagocytosis, Pkm2, retinal neovascularisation

## Abstract

Retinal neovascularisation (RNV) is manifested in various retinal pathological conditions, often leading to irreversible blindness. The oxygen‐induced retinopathy (OIR) mouse model proves to be a useful tool for understanding RNV pathogenesis. In this model, retinal vascular phenotype undergoes two distinct stages: neovascular formation, followed by spontaneous regression. While microglial functions in the neovascular formation stage have been extensively studied, their behaviors and roles during regression remain unclear. In this study, we characterise the spatiotemporal dynamics and molecular heterogeneity of retinal microglia across both stages. During RNV formation, microglia exhibit an outer‐to‐inner and central‐to‐midperipheral migration pattern, whereas a reversed migration trend is observed during regression. We confirm a highly glycolytic microglia (HGM) subpopulation during RNV formation and demonstrate its pro‐angiogenic role by targeting a highly expressed pyruvate kinase M2 (Pkm2), a crucial enzyme for glycolysis. Importantly, we find that microglia exhibit enhanced phagocytic activity during regression, constituting a distinct phagocytosis‐associated microglia (PAM) subtype, expressing mannose receptor C‐type 1 (Mrc1/CD206). Altogether, our findings reveal stage‐specific microglial functional dynamics, providing novel insights into RNV pathogenesis and intervention.

## Graphical abstract

In the oxygen‐induced retinopathy (OIR) mouse model, two distinct microglial subpopulations play opposing roles: highly glycolytic microglia (HGM) promote neovascular formation via Pkm2, while Mrc1/CD206‐positive phagocytosis‐associated microglia (PAM) facilitate its regression. This finding may provide a dual therapeutic strategy based on selectively inhibiting HGM while promoting PAM function.

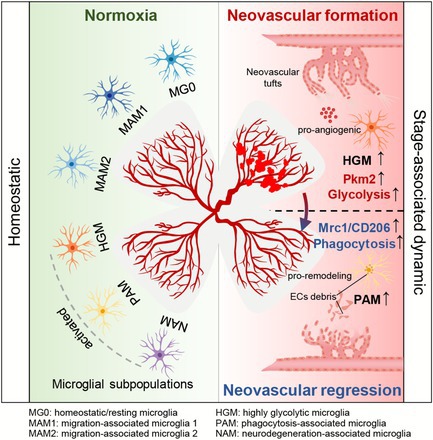

## 1 Introduction

Retinopathy of prematurity (ROP), retinal vein occlusion (RVO), and proliferative diabetic retinopathy (PDR) are among the leading causes of blindness worldwide, primarily due to retinal neovascularisation (RNV), characterised by the aberrant growth of structurally and functionally defective blood vessels in the retina [1, 2]. Elevated retinal vascular endothelial growth factor (VEGF) levels are a major driver of RNV; accordingly, anti‐VEGF therapy is widely used in clinical practice. However, some patients require repeated intravitreal injections, which increases the risk of ocular and systemic complications [3], while others show limited or no responsiveness [4]. These limitations highlight the urgent need for alternative interventions informed by a deeper mechanistic understanding of RNV pathogenesis.

Microglia, the resident immune cells of the retina, play essential roles in both vascular development and pathological angiogenesis [5, 6]. During early retinal development, microglia migrate into the ganglion cell layer (GCL) before vascularisation [7]. As vessels form, they infiltrate the outer plexiform layer (OPL), closely associate with and guide endothelial tip cells' outgrowth. In the mature retina, microglia are predominantly ramified and evenly distributed, with only a small proportion near vessels. Under RNV conditions, however, they become activated, adopting a hypertrophic amoeboid morphology with locally increased density, particularly around neovascular tufts [8]. Some geographical features in microglial distribution have also been reported during neovascular formation and revascularisation of avascular areas in oxygen‐induced retinopathy (OIR) mouse retinas [9]. However, a comprehensive view of the spatiotemporal dynamics of microglia, particularly encompassing the RNV regression phase, has yet to be established.

Consistent with their context‐dependent dynamics, microglia also exhibit substantial molecular heterogeneity [10]. Transcriptomic analyses have classically categorised them into three populations: resting microglia (M0), which specifically express TMEM119, P2Y12, and HEXB; pro‐inflammatory microglia (M1), which express CD16, iNOS, IL‐1β, and TNF‐α; and anti‐inflammatory microglia (M2), which are generally identified by CD206, Arginase‐1, and Ym1 [11]. These phenotypes are associated with distinct metabolic programs: M0 and M2 microglia preferentially generate energy via oxidative phosphorylation (OXPHOS) [12], whereas M1 microglia shift towards a glycolysis‐dominant metabolic profile [13]. Recent studies indicate that microglial heterogeneity is more complex than previously recognised [14]. Beyond the three classical populations, additional subsets have been identified in association with neurological disorders, including Alzheimer's disease, Parkinson's disease, and gliomas [15, 16, 17, 18]. In OIR mouse models, several subpopulations have also been reported, including RIP3‐positive microglia [19], RMG1 [20], and proliferative retinopathy‐associated microglia (PRAM) [21]. While these subtypes play important roles in the formation of pathological vessels, the molecular heterogeneity of microglia during the regression stage of RNV remains unknown. Elucidating the molecular features of microglia during the spontaneous RNV regression is crucial, as this knowledge could uncover novel therapeutic strategies to halt or reverse the disease progression.

To obtain a comprehensive view of microglial molecular and functional features, in this study, we conducted an in‐depth analysis of microglial spatiotemporal dynamics and subpopulation profiles during both RNV formation and regression. We characterised global microglial migration patterns across these stages and, through single‐cell RNA sequencing (scRNA‐seq), confirmed the presence of previously reported highly glycolytic microglia (HGM) involved in RNV formation. Moreover, we identified a novel phagocytosis‐associated microglia (PAM) subpopulation pertaining to RNV regression. These results combined demonstrate stage‐associated microglial dynamics that had previously been only partially understood.

## 2 Results

### 2.1 Microglial Migration and Homing Across Retinal Layers Coincide With RNV Formation and Regression

**FIGURE 1:**
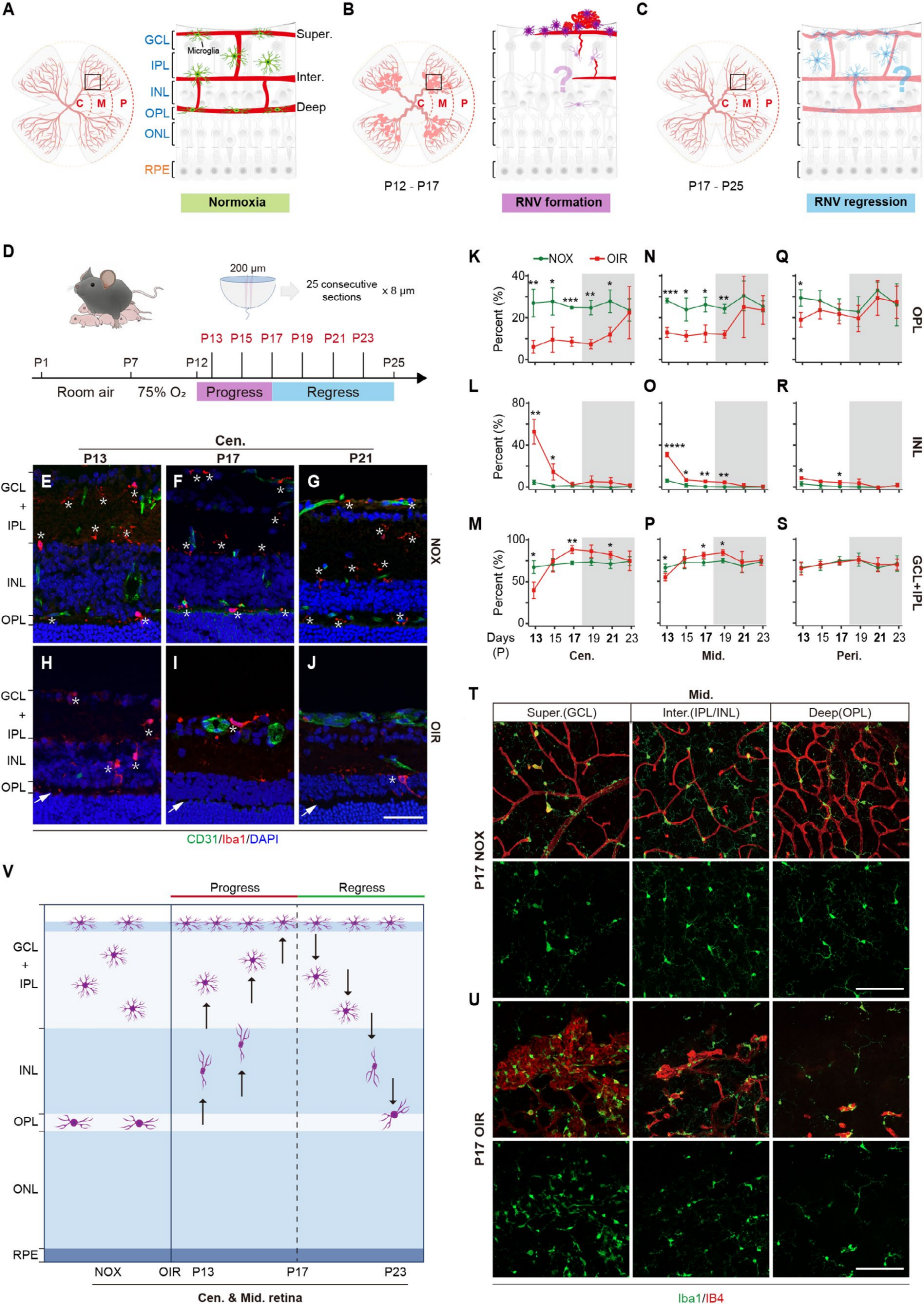
Microglial migration and homing during RNV formation and regression in the OIR mouse model. (A–C) Schematic illustration of retinal vasculature and microglia in the mouse retina. (A) NOX mouse retinas. The boxed area is a representative midperipheral retinal region, which is schematically illustrated as a vertical retinal section next to it. Microglia (light green) are distributed as three tiers along vessels (red) in normoxia retina. C, central; GCL, ganglion cell layer; INL, inner nuclear layer; IPL, inner plexiform layer; M, midperipheral; ONL, outer nuclear layer; OPL, outer plexiform layer; P, peripheral; RPE, retinal pigment epithelium. Inter., intermediate; Super., superficial; Deep. (B) P12‐P17 OIR retinas. Illustrated is the boxed area with RNV pathological vessels located on the superficial retinal layer with accumulated microglia (purple) and uncharacterized deep‐layer microglia (light purple). (C) P17‐P25 OIR retinas with RNV under regression. The illustrated retinal area shown in retinal section represents pathological vessels (light red) with microglia (light blue) under recovery. (D) Streamline of OIR mouse model creation and sample collection. Neonatal pups were exposed to 75% oxygen (O₂) at P7 and returned to room air at P12. Neovascularization peaks at P17, spontaneously regresses, and stabilises by P25. Twenty‐five consecutive sections across the optic nerve head for each eye were collected at 8 μm thickness each. (E–J) Immunofluorescence images of CD31‐labelled endothelial cells (green) and Iba1‐labelled microglia (red) from central regions of NOX (E–G) and OIR (H–J) retinas at P13, P17, and P21. DAPI is blue. Asterisks indicate numbers and locations of microglia in OIR and NOX retinas. Note the lack of microglia in OIR OPL indicated by arrows. Scale bar, 50 μm. (K–S) Quantification of the percentage of total Iba1^+^ cells located in the central (Cen.) (K, L, M), midperipheral (Mid.) (N, O, P), and peripheral (Peri.) (Q, R, S) retinas from P13 to P23. Green and red lines represent NOX and OIR retinal layers, respectively. The grey shaded areas represent the regression phase. *n* = 3 mice per group per time point, 25 sections/eye. Statistics was performed using two‐tailed unpaired Student's *t*‐test. *p < 0.05, **p < 0.01, ***p < 0.001, ****p < 0.0001. (T, U) Representative images of retinal flat mounts at P17 from NOX (T) and OIR (U) mice, stained for microglia (Iba1, green) and vasculature (IB4, red). Three layers of retinal vasculature are presented from left to right. Scale bars, 100 μm. (V) Schematic diagram of proposed microglial migration trajectory during RNV formation and regression in the central and midperipheral retina based on observations.

Under normoxic conditions, microglia are mainly localised to the GCL, inner plexiform layer (IPL), and OPL, and are almost absent from the inner nuclear layer (INL) (Figure 1A). In the OIR mouse model, microglia are known to concentrate around the superficial abnormal vasculature; however, their distributions across other retinal layers have remained largely unknown (Figure 1B). Meanwhile, it remains unclear whether microglial distribution returns to normal in the regression stage (Figure 1C). To address these questions, we established an OIR mouse model following a standard protocol [22] (Figure 1D) to carefully examine microglial spatiotemporal localisation during both RNV formation and regression.

We first examined the central retina of OIR mice, which experienced the highest oxygen shock (Figure 1E–J). Compared to the normoxic retinas, OIR retinas exhibited ectopic microglia labelled by Iba1 in the INL at postnatal day (P) 13, often extending elongated vertical processes towards the GCL (Figure 1H, asterisks). This phenomenon was transient, as these cells were nearly absent at P17 (Figure 1I), but reappeared at P21, frequently with elongated processes towards the OPL (Figure 1J, asterisk). Notably, a pronounced reduction of microglia within the OPL was consistently found in OIR retinas at all three time points (Figure 1H–J, arrows).

To further elucidate the relationship between microglial spatiotemporal dynamics and the RNV process, we quantified microglial distribution across retinal layers and along the central‐to‐peripheral orientation at different stages of RNV (Figure 1K–S). A significant reduction of microglia was consistently observed in the OPL of central retinas during RNV formation (before P17) (Figure 1K, red lines). Meanwhile, microglial fractions in the INL appeared to be higher than the normoxia (NOX) levels between P13 and P17, before gradually returning to normal levels by P17 (Figure 1L). During RNV regression (after P17), a small decrease in microglia within the GCL + IPL and an increase in the INL were observed (Figure 1L,M). Concomitantly, microglia in OPL nearly returned to normal levels (Figure 1M). Similar, though less pronounced, changes in microglial distribution were also observed in midperipheral retinas (Figure 1N–P). By contrast, microglial distribution showed little change in the peripheral retinas throughout both the formation and regression stages of RNV (Figure 1Q–S).

We further examined OIR retinal flat mounts, focusing on midperipheral retina areas where prominent RNV and microglial aggregation were evident in the superficial vascular plexus (approximately the GCL) at P17 (Figure 1T,U, left column). Microglial density in the intermediate vascular layer (between IPL and INL; IPL/INL) showed little change (Figure 1T,U, middle column), whereas a marked reduction in microglia was observed in the deep vascular layer (approximately the OPL) (Figure 1T,U, right column). These findings were consistent with our observations from retinal sections. Collectively, the above data suggest a predominant outer‐to‐inner microglial migration, especially in the central and midperipheral retinas, during RNV progression, followed by reverse‐direction homing during regression (Figure 1V).

### 2.2 Central‐To‐Midperipheral Microglial Dynamics Revealed by Three‐Dimensional Analysis of Flat‐Mount OIR Mouse Retinas

**FIGURE 2:**
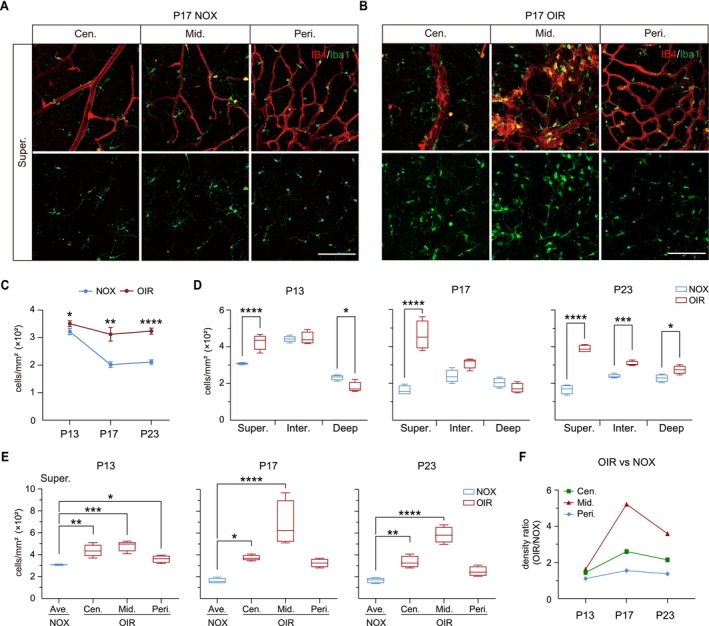
Horizontal microglial distribution on flat‐mount retinas. (A, B) Representative images of superficial vascular plexus layer of P17 NOX (A) and OIR (B) retinal flat mounts, showing Iba1‐labelled microglia (green) and IB4‐labelled (red) vessels in central (left), midperipheral (middle) and peripheral (right) retinas. Scale bars, 100 μm. (C) Quantification of the average microglial density across three retinal layers per unit area (mm^2^) from NOX and OIR flat mounts at P13, P17 and P21 (average three images/layer/retinal region/mouse, *n* = 4 mice). Statistics, Student's *t*‐test. (D) Box plots displaying microglial density of each NOX and OIR retinal layer at P13, P17 and P21. (E) Box plots of microglial density of central, midperipheral and peripheral superficial retina at P13, P17 and P21. Note that microglial density in NOX retinas was presented as an average (Ave.) of central, midperipheral, and peripheral superficial retina due to the microglial even distribution in the three layers, based on Iba1 staining. (D, E) Statistical significance was determined by two‐way ANOVA with Sidak's post hoc test. *n* = 4 mice, average three images/layer/retinal region/mouse. (F) The density ratio of OIR to NOX microglia in the superficial‐ layer from central, midperipheral, and peripheral regions. *p < 0.05, **p < 0.01, ***p < 0.001, ****p < 0.0001.

We next examined whether microglial distribution along the central–peripheral axis was altered in OIR retinas. At P17, analysis of the superficial layer revealed increased microglia in both central and midperipheral regions of OIR retinas, in contrast to the uniform distribution seen in NOX controls (Figure 2A,B). We then assessed the microglial density across the entire retina at P13, P17, and P23, corresponding to the initiation, peak, and regression stages of RNV, respectively. In NOX retinas, total retinal microglial density declined sharply from P13 to P17, probably due to the retinal growth over time [23], and remained stable at P23 (Figure 2C). In contrast, OIR retinas exhibited only a slight reduction over the same time frame.

To obtain a more detailed spatial profile, we surveyed microglia within each retinal layer along the central–peripheral axis. Across all three ages examined, OIR retinas consistently displayed a significant increase in the superficial layer, with only minor changes in the intermediate and deep layers (Figure 2D). We therefore subdivided the superficial layer into central, midperipheral, and peripheral regions to pinpoint the epicentre of the microglial response. In contrast to the relatively uniform microglial distribution in NOX retinas across these regions at all ages (Figure S1A–I), OIR retinas showed significantly increased microglial density in both central and midperipheral regions, whereas the peripheral region remained largely unchanged (Figure 2E and Figure S2A–I). Notably, the increase in microglial density was significantly greater in the midperipheral region than in the central region (Figure 2E). Quantitative analysis of fold change relative to the age‐matched NOX controls confirmed this observation, which was that midperipheral superficial retina exhibited the most dynamic microglial changes during OIR (Figure 2F).

### 2.3 Identification of Microglial Subpopulations in NOX and OIR Mouse Retinas by scRNA‐Seq

The above data demonstrate that OIR microglia are highly dynamic in spatiotemporal distribution. We went on to investigate their population heterogeneity in response to the RNV process. Microglia constitute only 1% of the retinal cell population [24]. RNV in the OIR mouse model peaked at P17 and declined mostly at P21 [25]. We isolated and enriched microglia from retinas of the above two time points using CD11b antibody‐conjugated microbeads and performed scRNA‐seq using the 10X Genomics platform (Figure S3A). Among 23,949 cells that passed the quality control (see Section 4 and Figure S3B–D), we identified 25 clusters, which we then assigned to 13 major cell types based on the expression of specific marker genes (Table S1 and Figure S3E–G). Within this comprehensive atlas, our enrichment strategy successfully captured 2936 CD11b‐positive (CD11b^+^) cells (~12% of total), which included distinct populations of microglia, monocytes, macrophages, and neutrophils (Figure S3F,G).

**FIGURE 3:**
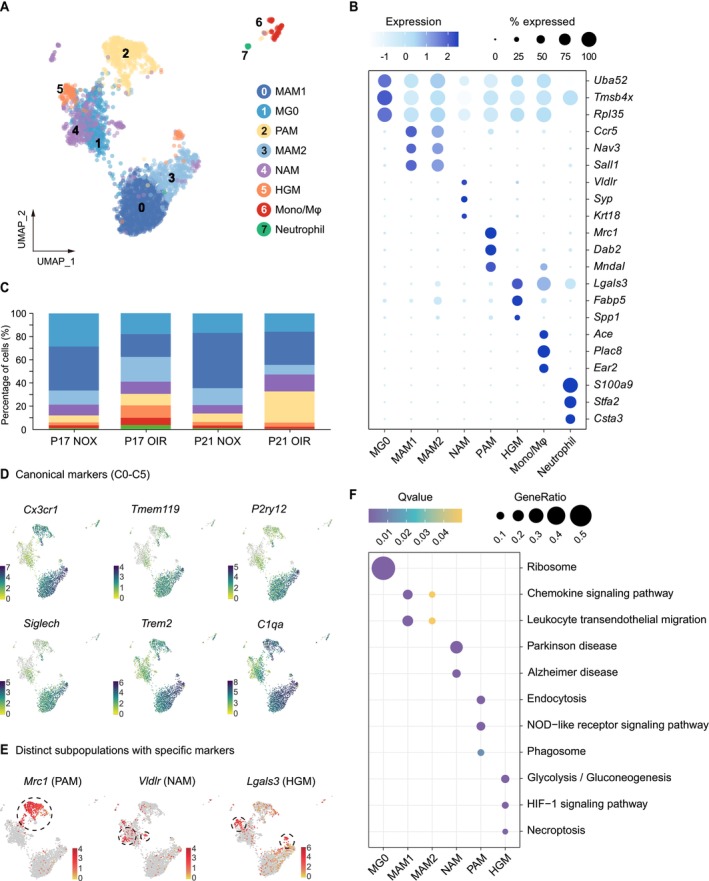
Single‐cell transcriptomic analysis of microglia heterogeneity. (A) Re‐clustering of CD11b^+^ cells in both NOX and OIR mouse retinas at P17 and P21. Eight distinct cell clusters (C0‐7) were defined: the resting microglia (MG0), migration‐associated microglia 1 and 2 (MAM1, MAM2), phagocytosis‐associated microglia (PAM), neurodegeneration‐associated microglia (NAM), highly glycolytic microglia (HGM), monocytes/macrophages (Mono/Mφ), and neutrophils. (B) Dot plotting expression of selected marker genes used to define each cell cluster. (C) Stacked bar plot showing the proportional abundance of each cell cluster in retinas from NOX and OIR mice at P17 and P21. (D) UMAP feature plots showing the expression of canonical microglia genes across subpopulations. (E) UMAP feature plots demonstrating expression of key marker genes associated with PAM (*Mrc1*), NAM (*Vldlr*), and HGM (*Lgals3*). Dashed circles indicate the corresponding subsets. (F) KEGG pathway enrichment by clusterProfiler.

To further study the microglia population, we re‐clustered the CD11b^+^ cells and identified eight transcriptionally distinct subpopulations (Figure 3A) based on gene expression profiles (Figure 3B). All eight subpopulations were found in both NOX and OIR retinas but with different proportions (Figure 3C). Six clusters (C0–C5) corresponded to microglia, one cluster (C6) represented a monocyte/macrophage (Mono/Mφ) population, and one cluster (C7) consisted of neutrophils. All microglial subpopulations expressed canonical markers such as *Siglech*, *P2ry12*, *Cx3cr1*, and *C1qa* (Figure 3D), although their expression was lower in C2, C4, and C5. Notably, these three clusters were relatively understudied and displayed unique marker signatures: C2 selectively expressed *Mrc1* (see later for details), whereas C4 and C5 were characterised by expression of *Vldlr* and *Lgals3* (Figure 3E).

To determine the functional identities of each cluster, we next performed KEGG pathway enrichment analysis on their respective marker genes using the clusterProfiler package [26] (Figure 3F). The C1 cluster exhibited a homeostatic/resting signature without activation‐associated markers and was therefore designated as MG0 [27]. C0 and C3 shared enrichment in chemokine signalling and leukocyte migration pathways and were termed MAM1 and MAM2 (migration‐associated microglia 1 and 2), respectively. C2 expressed endocytosis‐related markers (*Mrc1*, *Vcam1*, *Cd163*, *Ccl7*) and was classified as PAM. C4 displayed high expression of *Vldlr*, *Atf4*, and *Rora*, with pathway enrichment linked to neuroinflammation and neurodegeneration, thus categorised as NAM (neurodegeneration‐associated microglia). Finally, C5 was enriched for glycolysis‐related genes (*Fabp5*, *Pgk1*, *Pkm*, *Pgam1*) and was designated as HGM.

### 2.4 Phasic Microglial Trajectories During Neovascular Formation and Regression

**FIGURE 4:**
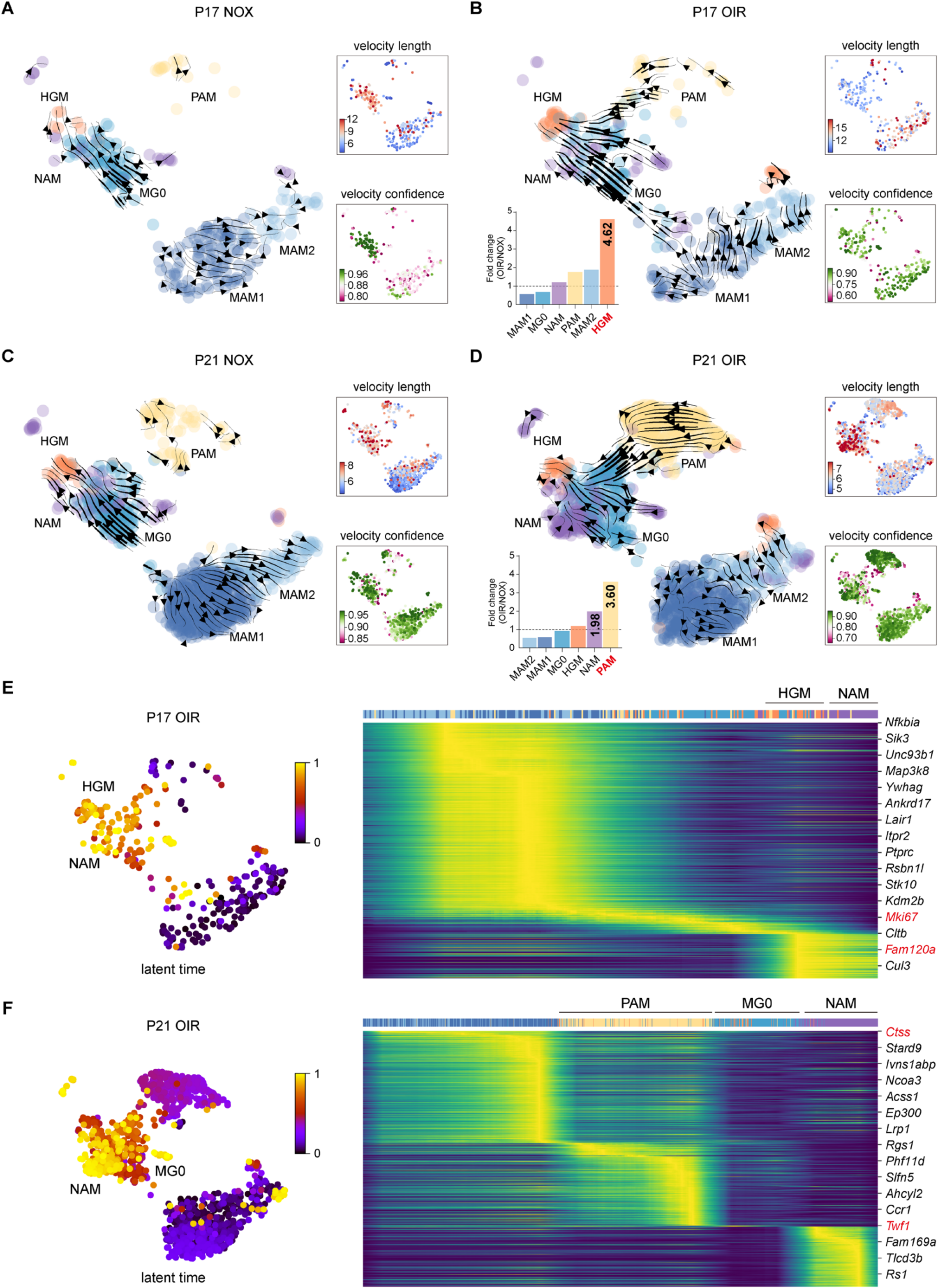
RNA velocity assessment of microglial trajectories during neovascularization and regression. (A–D) RNA velocity vectors projected onto UMAP embeddings, where similar vector directions indicate collective state transitions between subpopulations, and vector thickness reflects transition strength. Velocity length represents rates of transcriptional changes. Velocity confidence: Confidence in velocity length. (A) Microglial trajectory in P17 NOX retinas. (B) Microglial trajectory in P17 OIR retinas. The left‐corner inset shows proportional ratio of each microglial subpopulation in the OIR group to the NOX group. (C) Microglial trajectory in P21 NOX retinas. (D) Microglial trajectory in P21 OIR retinas. The left‐corner inset shows proportional ratio of each microglial subpopulation in the OIR group to the NOX group. (E, F) Heat map of the top 300 latent time‐dependent rank genes, showing gradual gene expression changes along the latent time trajectory.

We next investigated differences in the interconnection dynamics of microglial subpopulations between OIR and NOX retinas by performing RNA velocity analysis with scVelo using the *dynamical* model at P17 and P21—two time points that approximately correspond to the neovascularisation and regression stages (see Section 4). In the velocity map of a UMAP projection for the P17 NOX group, microglial subpopulations formed three distinct entities, HGM/NAM/MG0, MAM1/MAM2, and PAM (Figure 4A). In contrast, the OIR groups displayed overlapping velocity vector flows without clear boundaries (Figure 4B), suggesting dynamical transitions between subpopulations. Specifically, the vector trajectories indicated an MAM1 → MG0 → HGM transition (Figure 4B). Consistently, a proportional increase in HGM accompanied by decreases in MG0 and MAM1 was observed (Figure 4B, inset graph).

At P21, NOX microglia displayed velocity trajectories similar to those at P17 (Figure 4C compared to Figure 4A), both containing three discrete entities. However, the P21 OIR group lost the MAM1 → MG0 → HGM transition trajectory seen at P17 and instead exhibited a pronounced PAM → MG0 transition (Figure 4D). This group also showed part of the MG0 vectors were oriented towards NAM, with velocity length and confidence suggesting that PAM, NAM, and MG0 were highly dynamic (Figure 4D). Accordingly, the OIR group showed increased proportions of PAM (~3.60‐fold) and NAM (~1.98‐fold) (Figure 4D, inset graph).

To understand how microglial subpopulations evolved, we performed latent‐time analysis. In OIR retinas, NAM and HGM microglia emerged later than other subpopulations at P17 (Figure 4E), whereas MG0 and NAM microglia emerged late at P21 (Figure 4F). Analysis of the top 300 rank genes associated with estimated latent time revealed distinct transitional patterns among microglial subpopulations. Among the proteins encoded by these genes, MKI67 is a hallmark of cell proliferation; FAM120A interacts with NLRP3 inflammasome proteins and regulates inflammasome activation [28]; CTSS, preferentially expressed in mononuclear phagocytic cells, participates in MHC class II‐mediated antigen presentation [29]; and TWF1 has been implicated in macrophage‐mediated tumour immune infiltration [30]. These genes may play pivotal roles in orchestrating microglial subpopulation transitions during neovascularisation and regression.

### 2.5 HGM Glycolysis Is Essential for Neovascularisation

**FIGURE 5:**
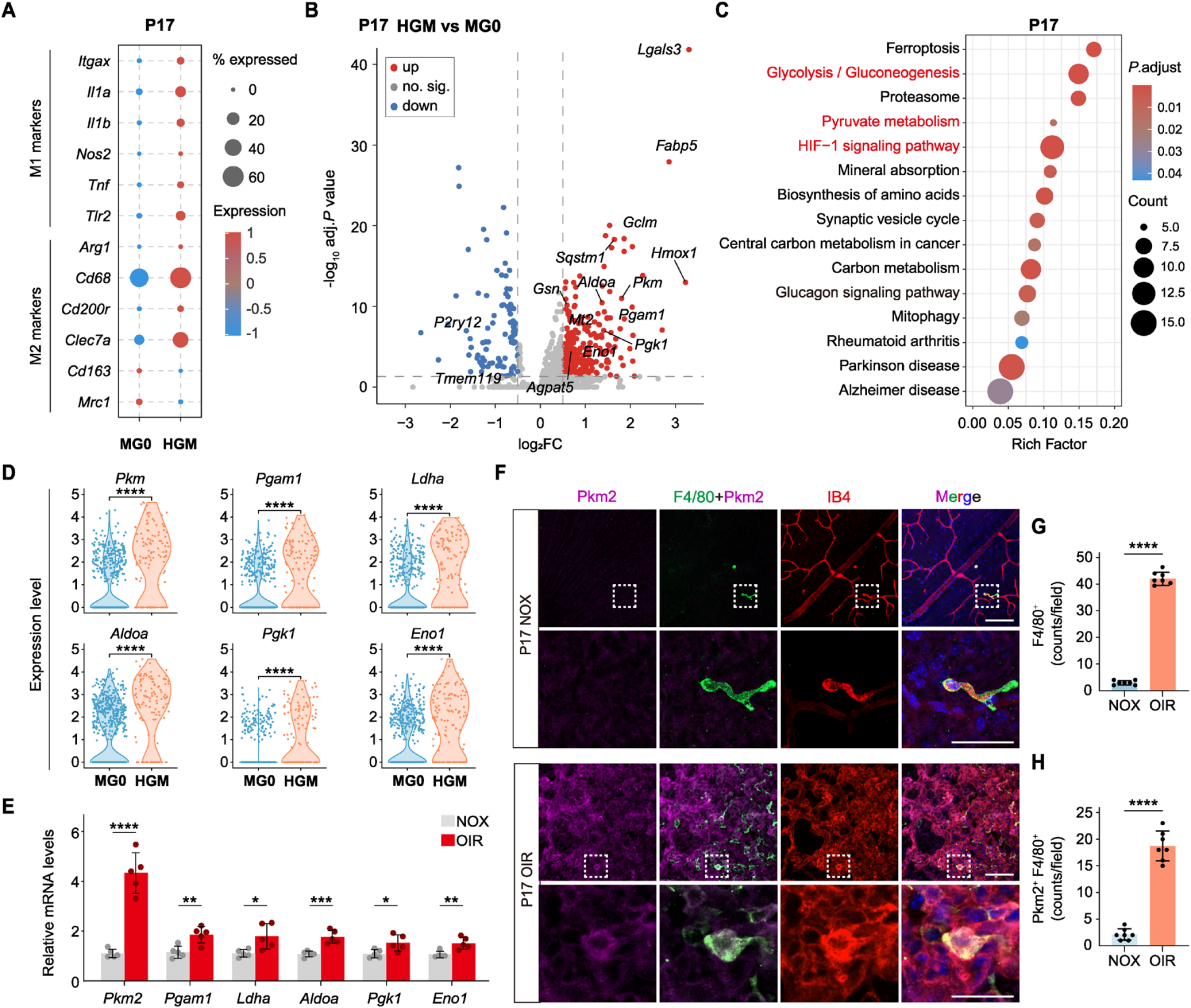
Association of Pkm2‐high HGM with pathological neovascularization. (A) Differential expression of M1 and M2 marker genes in P17 HGM and MG0 subpopulations. (B) Volcano plot demonstrating differentially expressed genes (DEGs) between HGM and MG0 subpopulations. Genes with |log_2_FC|(HGM vs. MG0) > 0.5 and adjusted *p* value < 0.05 are considered as significantly altered genes. Upregulated, red; downregulated, blue. (C) KEGG pathway enrichment of HGM upregulated genes compared to MG0. (D) Violin plots of six upregulated genes associated with glycolysis in HGM. (E) RT‐qPCR confirmation of HGM‐upregulated genes using isolated microglia with two‐step sequential purification using CD11b‐MACS (Magnetic Activated Cell Sorting). *n* = 5 mice. Statistical significance was determined by Student's *t*‐test. (F) Immunofluorescence images of Pkm2 stained (violet) flat‐mount retinas showed an elevated expression in OIR microglia (F4/80, green). Vasculature was labelled with IB4 (red), and nuclei were labelled with DAPI (blue). Scale bars, 50 μm (top), 100 μm (bottom). (G) Quantification of F4/80‐labelled microglia in OIR and NOX retinas. (H) Quantification of Pkm2 and F4/80 double‐labelled microglia in OIR and NOX retinas. *n* = 7 mice per group. Statistical powers were detected by Student's *t*‐test. *p < 0.05, **p < 0.01, ***p < 0.001, ****p < 0.0001.

Given that the HGM population predominantly correlates with RNV by RNA velocity analysis, we specifically focused on P17 HGM. Previous studies have shown that macrophages/microglia can be polarised into two functional states: pro‐inflammatory (M1) and anti‐inflammatory (M2), each proposed to be involved in neovascularisation [31]. Interestingly, we found that HGM expressed classic markers of both M1 and M2 (Figure 5A), suggesting it could represent an uncharacterised intermediate state between M1 and M2.

To obtain further functional insights into HGM, we next examined P17 HGM scRNA‐seq data compared with the counterpart resting MG0. Volcano plot demonstrated downregulation of homeostatic genes including *P2ry12* and *Tmem119*, an indication of microglial activation (Figure 5B). Upregulated *Lgals3* and *Homx1* genes in HGM were also described by other groups in the P17 OIR microglia population [19]. As expected, glycolysis‐associated genes were significantly upregulated compared to MG0, a hallmark of HGM identification (Figure 5B). KEGG enrichment of upregulated genes in P17 HGM showed metabolic processes including glycolysis/gluconeogenesis, pyruvate metabolism, and so forth (Figure 5C). Violin plots showed significantly increased expression levels of key glycolytic enzymes such as *Pkm*, *Pgam1*, *Ldha*, *Aldoa*, *Pgk1*, and *Eno1* in HGM microglia (Figure 5D). RT‐qPCR analysis further confirmed that HGM possessed high glycolytic activities (Table S2, Figure S4A,B and Figure 5E).

Among the glycolytic genes, *Pkm2*, a gene encoding a rate‐limiting enzyme in glycolysis, was upregulated most (Figure 5E). Consistently, elevated Pkm2 expression was detected by using immunofluorescence staining in P17 OIR retina (Figure 5F, violet), with increased F4/80‐labelled (F4/80^+^) microglia (green) associated with the neovasculature (Figure 5F,G). Quantification analysis confirmed increased Pkm2^+^/F4/80^+^ double‐positive microglia in OIR retinas (Figure 5H), some of which probably belonged to HGM.

**FIGURE 6:**
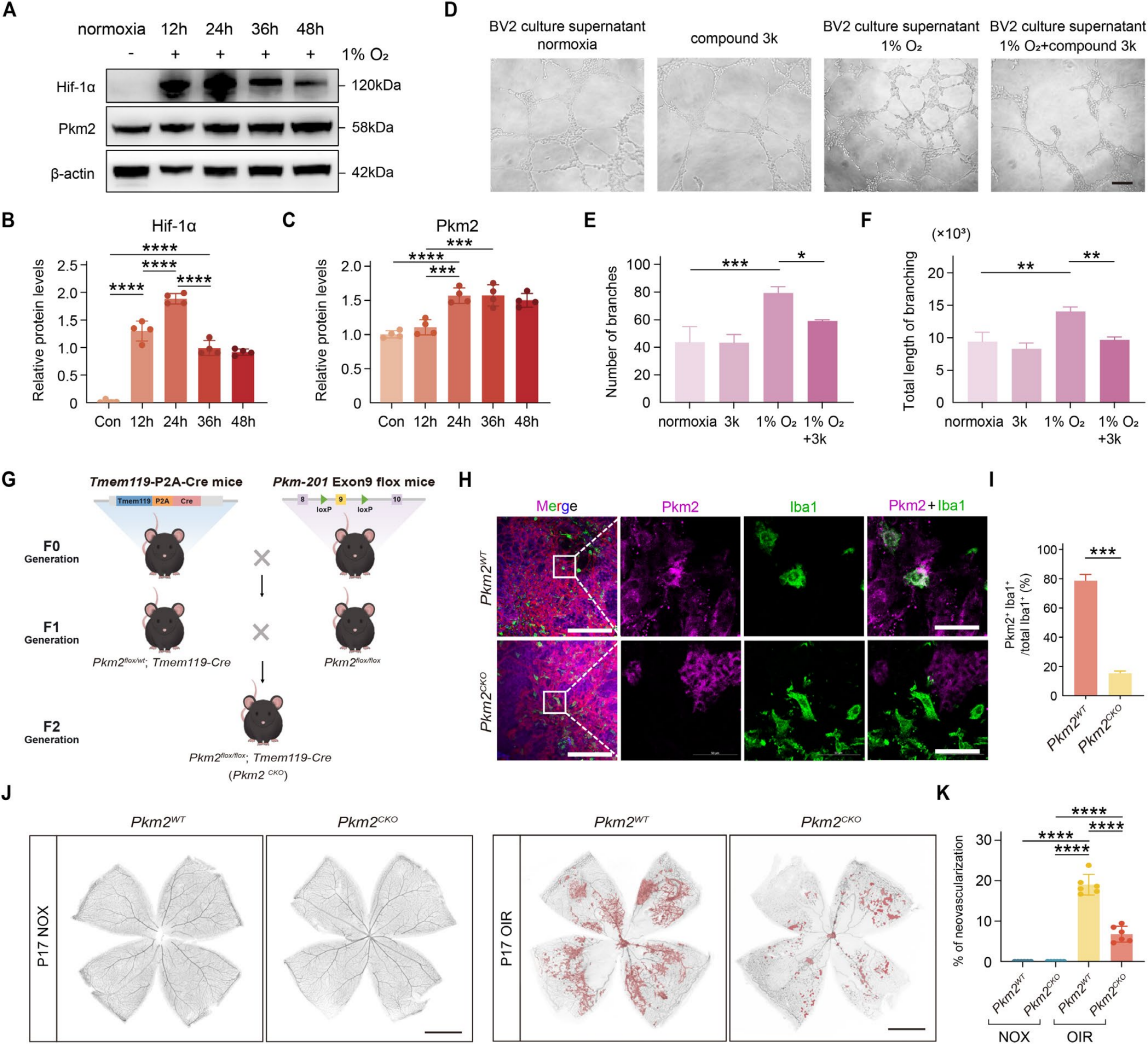
Pkm2 Inhibition ameliorates pathological retinal neovascularization. (A) Hif‐1α and Pkm2 expression detected by Western blotting in BV2 microglial cells cultured under normoxia (Con) and hypoxia (1% O₂) for 12, 24, 36, and 48 h. β‐Actin was used as an internal control. (B, C) Quantification of Hif‐1α (B) and Pkm2 (C) protein levels. The band intensities were normalized to that of β‐actin. *n* = 4. Statistical significance was determined by one‐way ANOVA followed Bonferroni test. (D) Tube formation assay of HUVECs treated with culture supernatant from BV2 cells under normoxia or hypoxia (1% O₂), with or without the Pkm2 inhibitor compound 3k (3k). Scale bar, 100 μm. (E, F) Quantification of the number of branches (E) and the total length of branches (F) from the tube formation assay shown in (D). *n* = 3. Statistical significance was determined by one‐way ANOVA followed Bonferroni test. (G) Schematic illustration of the breeding strategy for generating microglia‐specific *Pkm2* conditional knockout mice (*Pkm2* ^*flox/flox*^; *Tmem119*‐*Cre*, refers to as *Pkm2* ^*CKO*^) and their wild‐type littermate controls (*Pkm2* ^*flox/flox*^, refers to as *Pkm2* ^*WT*^). (H) Representative immunofluorescence images from *Pkm2* ^*WT*^ and *Pkm2* ^*CKO*^ flat‐mount retinas stained for Pkm2 (violet) and the microglial marker Iba1 (green). Note the remarkable reduction of Pkm2 in the *Pkm2* ^*CKO*^ microglia. Scale bars, 50 μm (main images), 20 μm (zoom in). (I) Quantification of the percentage of Pkm2‐positive microglia (Pkm2^+^ Iba1^+^) relative to Iba1^+^ cells. *n* = 7, two‐tailed Mann–Whitney test. (J) Inverted fluorescence images showing attenuated RNV formation in *Pkm2* ^*CKO*^ retinas compared with the wild type control. RNV vasculature was labelled by IB4 staining (highlighted in red). Scale bars, 1 mm. (K) Quantification of area with retinal neovascularization as a percentage of the total retinal area as shown in (J). *n* = 6 mice. Statistical significance was determined by one‐way ANOVA followed by Bonferroni test. *p < 0.05, **p < 0.01, ***p < 0.001, ****p < 0.0001.

To explore the impact of HGM on RNV, we assessed the suitability of BV2 cells as an in vitro model under hypoxic conditions. We first examined the expression of Hif‐1α and Pkm2, which are highly expressed in HGM (Figure 5B), in hypoxic BV2 cells. HIF‐1α and Pkm2 were both elevated within 24 h of culture (1% O_2_). However, Hif‐1α quickly declined while Pkm2 continuously increased over 48 h (Figure 6A–C). Increased cell death was observed after 24 h of hypoxia (Figure S5).

One of the potential mechanisms of microglia to promote pathological angiogenesis is via paracrine factors [32]. We therefore collected BV2 culture medium under hypoxia and treated human umbilical vein endothelial cells (HUVECs) to observe tube formation (Figure 6D–F). Culture supernatants from BV2 cells exposed to hypoxia for 24 h significantly promoted endothelial cell tube formation compared with the NOX group, as evidenced by increased branch numbers and branch lengths (Figure 6E,F). By contrast, culture medium from BV2 cells treated with the Pkm2 inhibitor compound 3k diminished the angiogenic effects (Figure 6E,F). Together, these data suggest that HGM may influence angiogenesis through a Pkm2‐dependent mechanism similar to that observed in BV2 cells.

To further elucidate the role of Pkm2‐rich HGM in pathological angiogenesis in vivo, we conditionally ablated *Pkm2* in microglia using a *Tmem119‐Cre* driver (*Pkm2* ^*flox/flox*^, referred to as *Pkm2* ^*WT*^; *Pkm2* ^*flox/flox*^; *Tmem119‐Cre*, referred to as *Pkm2* ^*CKO*^) (Figure 6G). The loss of Pkm2 expression was confirmed in Iba1‐labelled microglia (Figure 6H,I). OIR mouse models were generated in both *Pkm2* ^*CKO*^ and *Pkm2* ^*WT*^ mice, and RNV formation was assessed. A remarkable decrease of neovascular areas was observed in *Pkm2* ^*CKO*^ OIR retinas (Figure 6J,K). The data together strongly suggest that highly glycolytic HGM is a crucial driver of RNV.

### 2.6 Association of PAM With the Regression of Neovascularisation

**FIGURE 7:**
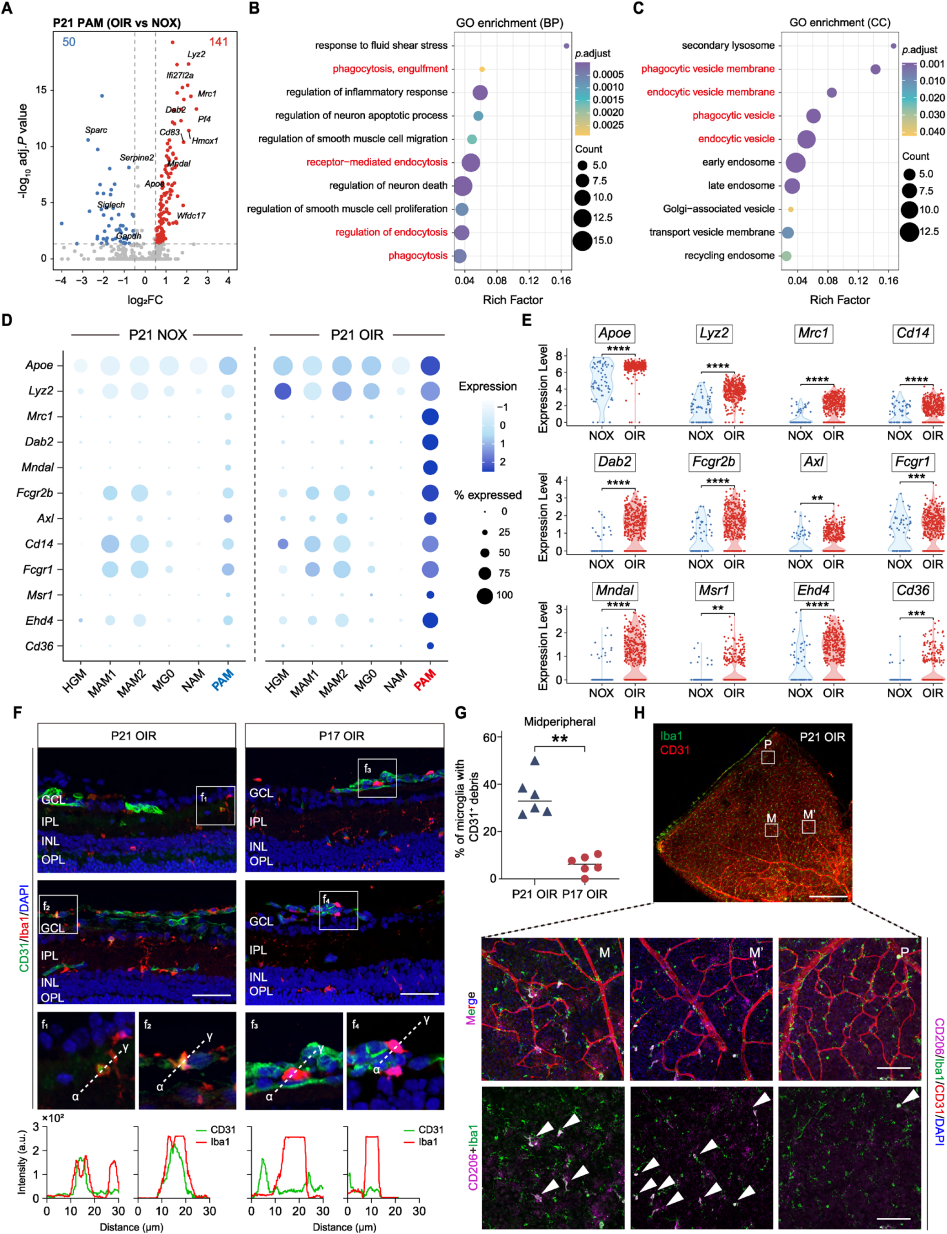
Association of PAM with RNV regression at P21. (A) Differentially expressed genes in P21OIR PAM relative to NOX group. Genes with |log_2_FC| > 0.5 and adjusted *p* value < 0.05 are considered to be DEGs. Up‐regulated genes in red, while down‐regulated in blue. (B, C) Gene ontology (GO) enrichment for the 141 upregulated genes. Dot plot depicting the top 10 enriched GO terms with the largest gene ratios for biological processes (B) and cellular components (C). The size of the dots represents the number of genes in the significant differentially expressed gene list associated with the GO term, and dots' colours represent the adjusted *p* values. (D) Average expression level and the percentage of expressing cells of phagocytosis‐associated genes across different subpopulations. (E) Violin plots comparing the expression levels of phagocytic‐associated genes in the PAM between the NOX and OIR groups at a single‐cell level. **p < 0.01, ***p < 0.001, ****p < 0.0001. (F) Co‐labelling of CD31‐positive endothelial cells (green) and Iba1 positive microglia (red) in P17 and P21 OIR retinas. Note the green signal was found within the microglial cell body (red) of P21 but not the P17 OIR GCL layer. DAPI staining indicates nuclei (blue). Scale bars, 50 μm. Fluorescence intensity profiles across the dashed lines in the images (bottom) showed relative localization of red (microglia) to green (endothelial cells) signals. (G) Quantification of the percentage of Iba1^+^ microglia containing CD31^+^ endothelial debris in the midperipheral retina of OIR mice at P21 and P17. Each data point represents the mean percentage calculated from three retinal sections passing through the optic nerve head per mouse. *n* = 6 mice, two‐tailed Mann–Whitney test. **p < 0.01. (H) A flat‐mount retina of P21 OIR mouse stained with Iba1 (green) and CD31 (red). Higher‐magnification views of the boxed areas are shown below. M and M′ were two fields from the midperipheral retinal region. P, peripheral region. The bottom panels display additional staining for the PAM marker Mrc1/CD206 (violet) from the same region. White arrowheads indicate CD206^+^ Iba1^+^ double‐positive cells. Scale bars, 500 μm (top panel), 100 μm (bottom).

We next investigated the functional properties of the PAM subpopulation, which was most abundant at P21 in OIR retinas (Figure 4D, inset). We first compared the gene expression profiles of PAM between OIR and NOX retinas at P21, focusing on the 141 genes upregulated in OIR PAM (Figure 7A). Gene ontology (GO) enriched terms predominantly related to phagocytosis and endocytosis in both the biological process (BP) and cellular component (CC) categories (Figure 7B,C). Examination of known phagocytic genes, such as *Apoe*, *Lyz2*, *Mrc1/CD206*, across all clusters showed overall higher PAM expression in both OIR and NOX groups (Figure 7D). At the single‐cell level, OIR PAM showed significantly elevated phagocytic gene expression (Figure 7E). At the tissue level, enhanced phagocytic activities were evidenced by the observation of CD31^+^ endothelial debris within microglial cell bodies in P21 OIR retinas (Figure 7F). Fluorescence intensity line profiles further confirmed the intracellular localisation of these endothelial remnants, indicating active engulfment (Figure 7F, bottom panels). Quantitative analysis demonstrated that the percentage of microglia containing CD31^+^ debris was significantly higher in P21 OIR retinas compared to P17 (Figure 7G). This increase coincides with the active vascular regression and remodelling occurring in the midperipheral retina at P21.

To further establish the correlation between the observed phagocytic cells and the PAM subpopulation, we performed immunohistochemistry for the PAM‐specific marker Mrc1/CD206, identified by our single‐cell analysis (Figure 3E). Consistently, we observed a distinct population of Mrc1/CD206^+^ Iba1^+^ microglia accumulating in the midperipheral region of P21 OIR retinas, where abnormal vessels were undergoing regression (Figure 7H, boxed regions M and M'). Conversely, Mrc1/CD206 expression was barely detectable in microglia within the peripheral regions characterised by relatively healthy vasculature (Figure 7H, boxed region P). These findings support a functional association between PAM and the clearance of pathological vasculature.

## 3 Discussion

RNV, a devastating complication manifested in many ischemic retinopathies, poses a formidable clinical challenge due to its persistence and progressive nature; it rarely spontaneously regresses. A large body of studies have focused on RNV formation, resulting in mounting mechanistic understanding of the disease pathogenesis [33]. In contrast, RNV regression has been largely understudied. The OIR mouse model is commonly used to study aspects of RNV, yet it uniquely exhibits spontaneous regression, which offers a unique opportunity to investigate both RNV formation and regression. In this study, we investigated microglial dynamics throughout the entire RNV course, offering a comprehensive view of microglial functions in vascular pathogenesis associated with specific stages.

Previous studies have illustrated microglial density and distribution in OIR neovascularisation, primarily focusing on superficial retinal layers, where neovascular tufts form under hypoxic induction. A consistent observation is the accumulation of microglia within the superficial neovascular tufts at P17, the peak of neovascularisation [8, 9]. We performed an in‐depth analysis of microglial distributions across retinal layers from central (avascular) to midperipheral (neovascular) to peripheral regions. Moreover, we undertook a denser temporal sampling at six time points spanning both RNV formation and regression. Our data suggest that during RNV formation, microglia undergo a stage‐associated intertwined outer‐to‐inner and likely central‐to‐midperipheral aggregation, which has not been described previously. These migratory microglia are likely to contribute to the growth of neovascular tufts on superficial retinas. Interestingly, as pathological vessels resolve, a reverse microglial migration trend—from inner to outer retinal layers—appears to occur. The differential microglial behaviours probably reflect their molecular and functional heterogeneity pertaining to different disease stages, either harmful or beneficial, which may provide clinical targeting insights.

The identification of six distinct microglial subpopulations underscores their high heterogeneity, each defined by a unique molecular profile, beyond the classical M1/M2 binary states [34]. The high glycolytic HGM subpopulation identified in this study is similar to the reported pathological retinal angiogenesis–associated glycolytic macrophages/microglia (PRAGMs) [35]. HGM is specific to RNV formation but not regression, with a profound glycolytic shift compared to the resting microglia, characterised by elevated Pkm2 expression. Consistent with the suggested role of HGM in neovascularisation, conditional ablation of *Pkm2* specifically in microglia attenuated the pathogenic phenotype in OIR mice. Notably, ablation of another glycolysis enzyme, PFKFB3, in myeloid lineages has achieved a similar effect [35]. Our results, combined with those of others, strongly support the notion that enhanced glycolytic activity is a conserved RNV driver.

One of our key findings is the identification of a phagocytic microglial subpopulation, PAM, which is specific to RNV regression. The enhanced expression of phagocytosis‐associated genes in this subtype coincides with the observed engulfed endothelial cell debris during neovascular resolution but not during formation. Furthermore, we are able to identify the accumulation of PAMs around the involuting vasculature by Mrc1/CD206, a faithful identifier of PAM. These results suggest that PAMs may play a beneficial role in remodelling pathogenic vasculature.

The stage‐association of microglial dynamics was further revealed by tracking developmental trajectories of microglial subsets at different RNV stages. RNA velocity analysis on microglia from both P17 and P21 implied a stage‐dependent reprogramming of microglial transition dynamics. At P17, during peak neovascularisation, we identified a clear transitional trajectory from migratory (MAM1) through resting (MG0) to HGM. Accordingly, significant expansion of HGM was found at the expense of MAM1 and MG0. Thus, further elucidating molecular pathways by which MAM1 or MG0 transition into HGM may provide opportunities to therapeutically modulate RNV.

RNA velocity analysis suggested at P17, HGM may arise from MG0. By the regression phase at P21, HGM returns nearly to NOX levels. An intuitive assumption would be that HGM reverses its trajectory back to MG0—a similar notion has been proposed in a previous study to describe relationships among microglial subpopulations [21]. However, RNA velocity indicated otherwise: during regression, it is the PAM that transitions towards MG0, part of which further transforms into neurodegeneration‐associated microglia, NAM. Further investigation into the potential connections between HGM and PAM in OIR retinas, particularly during the interval between P17 and P21, will be of considerable interest. Additionally, the MG0 → NAM route might underlie the long‐term retinal thinning and neuronal dysfunction known to persist in the OIR mouse model even after the vascular phenotype has recovered [36].

Although our dense spatiotemporal retinal sampling revealed novel aspects of microglial dynamics and heterogeneity, particularly in an RNV stage‐associated manner, and thus expanded prior knowledge, real‐time in vivo observation of microglia in RNV retinas will still be required with the implementation of genetic lineage tracing and two‐photon imaging technology. Besides, several key questions remain unanswered, including the mechanisms by which PAM participates in pathological vasculature remodelling and the processes underlying microglial subtype transitions, insights that will be essential for identifying therapeutic targets for RNV treatment.

## 4 Materials and Methods

### 4.1 Mouse Generation and Breeding

The use of animals followed the approved guidelines of the Animal Care and Use Committee of Zhongshan Ophthalmic Center and the Association for Research in Vision and Ophthalmology (ARVO). All experimental protocols were approved by the animal experimental ethics committee of Zhongshan Ophthalmic Center, Sun Yat‐sen University, China (authorised number: Z2024046). All mice, including experimental parental lines, were of C57BL/6J genetic background and were purchased from the GemPharmatech Co. Ltd., Jiangsu, China. The *Pkm2* ^*flox/flox*^ parental line was crossed onto *Pkm2* ^*flox/wt*^; *Tmem119*‐*Cre* parental line to generate *Pkm2* ^*flox/flox*^; *Tmem119*‐*Cre* (*Pkm2* ^*CKO*^, experimental) and *Pkm2* ^*flox/flox*^ (*Pkm2* ^*WT*^, control) litter mates. The *Tmem119*‐*Cre* transgene drives microglia‐specific *Cre* expression [37]. Animals were kept in a specific pathogen‐free (SPF) facility and maintained on an irradiated sterile diet with clean water under a 12 h light/dark cycle.

### 4.2 Establishment of the OIR Mouse Model

The OIR mouse model was established as previously described [22]. On postnatal day 7 (P7), newborn pups and their nursing mothers were exposed to 75% oxygen in a hyperoxia chamber for 5 days and then returned to room air (21%) at P12 to induce RNV, which peaks at P17 and subsequently undergoes spontaneous regression. At P17, mice weighing outside the 5.0–7.5 g range were excluded from all subsequent analyses to minimise variability [38]. Age‐matched mice kept in room air served as the NOX controls. At designated time points, mice were sacrificed by CO_2_, and their eyes were enucleated for further experiments. To quantify retinal pathology, an investigator blinded to the experimental conditions measured the areas of vaso‐obliteration (VO) and neovascularisation (NV) using Adobe Photoshop 2025. These values were then expressed as a percentage of the total retinal area.

### 4.3 Retinal Cryosections, Flat Mounts and Immunofluorescence Staining

Enucleated eyes were fixed in 4% paraformaldehyde (PFA) for 15 min, followed by a secondary fixation for 45 min after removing the cornea, iris, lenses, and vitreous.

For retinal cryosections, eyecups went through a 10%–30% sucrose gradient and were embedded in optimal cutting temperature (OCT) compound at −20°C overnight. A total of 25 consecutive sections spanning a 200 μm region encompassing the optic nerve head (ONH) were collected, with 8 μm thickness each. Sections were incubated with primary antibodies overnight at 4°C, followed by brief washes, and a subsequent 2 h incubation at room temperature with corresponding secondary antibodies.

For retinal flat mounts, isolated retinas were radially incised for flattening and incubated overnight at 4°C in the blocking buffer (1× PBS with 10% bovine serum albumin and 1% Triton X‐100). Retinas were subsequently incubated with primary antibodies in the blocking buffer at 4°C for 48 h, followed by long washes (3 × 30 min) and a subsequent 24 h incubation with corresponding secondary antibodies.

The primary antibodies are: rabbit anti‐Iba1 (1:400; Abcam; ab178846), goat anti‐Iba1 (1:400; Wako; 011‐27991), rat anti‐CD31 (1:400; BD; 557355), rat anti‐F4/80 (1:200; Abcam; ab6640), rabbit anti‐PKM2 (1:200; Cell Signalling Technology; 4053T) and rabbit anti‐CD206 (1:200; ServiceBio; GB113497). The secondary antibodies include donkey anti‐rabbit IgG H&L (Alexa Fluor 488, 568, 647) secondary antibody (1:1000; Invitrogen; A21206, A10042, A31573), donkey anti‐rat IgG H&L (Alexa Fluor 488) secondary antibody (1:1000; Invitrogen; A48269), donkey anti‐goat IgG H&L (Alexa Fluor 647) secondary antibody (1:1000; Invitrogen; A21447) and DAPI (1:3000; Roche; 10236276001). Additionally, the fluorescent‐conjugated Isolectin B4‐594 (1:400; Thermo Fisher Scientific; I21413) was also used to label the retinal vasculature.

### 4.4 Quantification of Microglia Distribution in Retinal Cryosections and Flat Mounts

Images of retinal cryosections and flat mounts were obtained using an AxioImager.Z2 microscope and an LSM 980 confocal microscope (both Zeiss, Germany). All images were processed using ZEN 3.4 software (Zeiss, Germany). The quantification of Iba1^+^ microglia was performed manually. Specifically, the retina was divided into three regions based on the average distance from the ONH to the retinal periphery edge: central: < 0.6 mm; midperipheral: 0.6–1.2 mm; and peripheral: > 1.2 mm.

For sections, the distribution of microglia within each retinal region and layer was expressed as cell percent: Iba1^+^ cells in the interested retinal area divided by the total number of Iba1^+^ cells of the entire image. For retinal flat mounts, we counted microglia within the three‐layer retinal vascular network—the superficial, intermediate, and deep layers [39]. Microglial density was given by the following calculation: Number of microglia within an imaging field divided by the field area, which is equivalent to 0.0784 mm^2^.

### 4.5 Single‐Cell Sample Preparation and Sequencing

Single‐cell RNA‐sequencing (scRNA‐seq) was performed for two different time points (P17 and P21) of OIR and NOX retinas. Mice were sacrificed by overdosing anaesthetics (1% pentobarbital sodium, 20 mL/kg). Twelve retinas from 6 mice for each group were collected and cut into small pieces before incubation in a 3 mL enzymatic hydrolysis solution containing 2 mg/mL papain (Worthington; LS003119), 1 mg/mL collagenase, Type I (Sigma–Aldrich; C0130), and 1 mg/mL DNase I (Roche; 10104159001). After enzymatic digestion at 37°C for 15 min, single‐cell suspensions were obtained and then filtered through a 30‐μm filter and cytocentrifugated in 300 × *g* for 7 min at 4°C. Cells were resuspended with 180 μL MACS (magnetic activated cell sorting) buffer (0.01 mM EDTA + 0.04% BSA + 1× PBS) and incubated with 20 μL CD11b Microbeads (Miltenyi; 130‐093‐634) at 4°C for 15 min. CD11b^+^ cells were subsequently isolated and enriched after passing through the MACS column and used to generate single‐cell gel bead‐in‐emulsion (GEM). ScRNA‐seq libraries were constructed using the Chromium Next GEM Single Cell 3′ Reagent Kits v3.1 according to the manufacturer's instructions and sequenced on the Illumina NovaSeq 6000 platform.

### 4.6 ScRNA‐Seq Analysis

After quality control and filtration of reads with low‐quality barcoded and unique molecular identifiers (UMIs), reads were mapped to the mouse genome using Cell Ranger software (10× Genomics, version 5.0.0). The digital gene expression matrix was used for downstream analysis in R (version 4.2.2) using the Seurat package (version 3.1). High‐quality cells were preserved under the following criteria: (1) the number of genes identified in a single cell ranged from 500 to 4000; (2) the total number of UMIs in a single cell was more than 500; (3) the mitochondrial gene percent in a single cell was less than 10%. Then PCA, UMAP, and louvain graph‐based clustering were performed for cell cluster and visualisation. Cell types were identified and annotated using SingleR based on their expression patterns.

### 4.7 Differential Expression and Enrichment Analysis

Differential gene expression was analysed using the FindMarkers function of Seurat. Genes with adjusted *p* < 0.05 and absolute log_2_ (Fold Change) > 0.5 were considered as differentially expressed genes (DEGs). These DEGs were subsequently used for GO and Kyoto Encyclopedia of Genes and Genomes (KEGG) enrichment analyses.

### 4.8 RNA Velocity

Single‐cell RNA velocity analysis was performed using scRNA‐seq data generated from the retinas from NOX and OIR mice. Initially, BAM files were preprocessed using samtools to ensure compatibility with velocyto.py. The Loom files generated by velocyto.py were subsequently processed using scVelo for downstream analysis [40]. Microglia were retained for further RNA velocity analysis. The moments of normalised spliced and unspliced counts were calculated for each cell and used to perform PCA analysis using the scvelo.pp.moments function, with default parameters applied for nearest neighbours and dimensions (n_pcs = 30). The RNA velocity was estimated using the scvelo.tl.velocity function with the ‘dynamical’ model, and the velocity graph was built using the scvelo.tl.velocity_graph function. For visualisation, the RNA velocity was projected into two‐dimensional uniform manifold approximation and projection (UMAP) embedding spaces using the scv.pl.velocity_embedding_stream function. Velocity length, velocity confidence, and latent time values were also overlaid onto these embeddings to highlight key patterns of transcriptional dynamics and progression of cellular states.

### 4.9 RNA Isolation and RT‐qPCR Analysis

MACS purification of CD11b^+^ cells, as described previously, was performed twice to ensure microglia purity. Purified cells were diluted using 1 × PBS to 300–400 cells/μL. Total RNA was subsequently extracted from the CD11b^+^ cells using the Single Cell Full‐Length mRNA Amplification Kit (Vazyme; N712) according to the manufacturer's protocol. qPCR was performed using qPCR Master Mix (Biomed; MT561). mRNA expression levels (CT values) were normalised to β‐actin (Actb) as follows: Fold change = 2^−∆∆CT^. Primer sequences for RT‐qPCR are listed in Table S2.

### 4.10 Cell Culture and Treatments

#### 4.10.1 BV2 Cells Culture

Mouse BV2 cells (CL‐0493) were obtained from Procell Life Science and Technology Co. Ltd. (Wuhan, China). BV‐2 cells were cultured in Dulbecco's modified Eagle's medium (DMEM) supplemented with 10% foetal bovine serum (FBS), 2 mM l‐glutamine, 100 mg/mL streptomycin, and 100 U/mL penicillin/streptomycin. Cells were cultured in a CO_2_ incubator at 37°C, 5% CO_2_.

#### 4.10.2 Human Umbilical Vein Endothelial Cells (HUVECs) Culture

HUVECs (CP‐H082) were obtained from Procell Life Science and Technology Co. Ltd. (Wuhan, China) and used between passages 3–5. HUVECs were cultured in HUVEC Cell Complete Medium (CM‐0122) with 100 U/mL penicillin/streptomycin. Cells were cultured in a CO_2_ incubator at 37°C, 5% CO_2_.

#### 4.10.3 BV2 Cells Treatment With Hypoxia and Pharmacological Pkm2 Inhibitor

BV2 were placed in a modular incubator chamber (Smartor 118, Hariolab) at 37°C with 1% O_2_ for different time periods (12, 24, 36, 48 h), then cells and culture supernants were collected for subsequent use. For Pkm2 inhibition, the BV2 cells were supplemented with Pkm2 inhibitor, compound 3k (150 nM in DMEM) for 24 h under either normoxic or hypoxic conditions.

#### 4.10.4 CCK8 Assay

To assess cell viability, BV2 cells were seeded onto 96‐well plates at a density of 1000 cells per well in 200 μL of complete medium and allowed to adhere for 24 h. Cells were then subjected to normoxic (21% O_2_) or hypoxic (1% O_2_) conditions for 12, 24, 36, and 48 h, respectively. At the end of treatment, 20 μL of Cell Counting Kit‐8 (CCK‐8) solution (CK04, DOJINDO) was added to each well of cells and incubated for an additional 4 h at 37°C. Light absorbance was then measured at 450 nm using a microplate reader. Because extensive cell death was observed after 24 h hypoxia treatment, we collected BV2 supernatant at 24 h's culture for tube formation (see below).

### 4.11 Tube Formation Assay

The in vitro angiogenic capacity of HUVECs was assessed using a tube formation assay on Matrigel (356230; Corning). One day prior to the experiment, HUVECs were serum‐starved for 24 h in DMEM supplemented with 0.2% FBS. Matrigel was dissolved overnight at 4°C.

The next day, 50 μL Matrigel was added to each well of a pre‐cooled 96‐well plate and incubated at 37°C for 30 min to allow the gel to polymerise. The serum‐starved HUVECs were harvested and resuspended, and a cell suspension of 10 μL containing 2–3 × 10^4^ cells was subsequently seeded onto the surface of the Matrigel. Tube formation under different treatment conditions was observed after incubating for 12 h at 37°C. HUVECs were supplemented with (1) Normoxia BV2 culture supernatant; (2) Compound 3k only; (3) 1% O_2_ hypoxic BV2 culture supernatant; (4) Compound 3k + 1% O_2_ hypoxic BV2 culture supernatant. The extent of tube formation in the above four groups was assessed by quantification of the total tube length and number of junctions using ImageJ software (NIH).

### 4.12 Western Blotting

After treatments, BV2 cells from each group were collected, and their total proteins were extracted using RIPA lysis buffer added with protease inhibitors (Sigma Aldrich, P7626). After bicinchoninic acid (BCA) protein assay, equal amounts of the protein (40 μg) from each group were loaded for sodium dodecyl‐sulphate polyacrylamide gel electrophoresis (SDS‐PAGE) and transferred onto a polyvinylidene fluoride (PVDF) membrane. The PVDF membrane was blocked using Tris‐buffered saline (TBS) containing 5% skim milk and 0.1% Tween‐20 (TBST) at RT for 1 h. Primary antibodies (Hif‐1α, 36169S; Pkm2, 4053T, and β‐actin, 4970S, Cell Signalling Technology) were then added to the blocking buffer (1:1000) and continued incubating overnight at 4°C. The PVDF membrane was washed with TBST and incubated with horseradish peroxidase (HRP)‐conjugated secondary antibody at 1:3000 for 2 h at RT. After washes with TBS, chemiluminescent signal from the HRP‐catalysed substrates' reaction (Thermo Scientific, 34580) was imaged and band densitometry was analysed using Image J software (NIH). Protein expression levels were first normalised to the β‐actin signal, then to controls, and expressed as fold changes.

### 4.13 Statistical Analysis

All quantitative data are presented as mean ± standard deviation (SD). Statistical analyses were conducted using GraphPad Prism 9.5.0. The normality of all data was first confirmed by the Shapiro–Wilk test (*p* > 0.05). For comparisons between two groups, a two‐tailed unpaired Student's *t*‐test or Mann–Whitney test was applied. For multiple groups, a one‐way or two‐way analysis of variance (ANOVA) was used, followed by Bonferroni's post hoc correction when appropriate. A two‐sided *p* value of less than 0.05 was considered statistically significant. The statistical significance was denoted as: *p < 0.05, **p < 0.01, ***p < 0.001, ****p < 0.0001, and ns means no significance.

## Author Contributions

Tao Li, Chunqiao Liu and Yuan Ma designed the study. Yuan Ma, Ziye Chen, Baoyi Liu, Jizhu Li, Lan Jiang, Zitong Chen, Yuan Liu and Xiaoyue Wei performed experiments. Zhuojun Xu, Zhuangling Lin, Chinling Tsai, Rebiya Tuxun, Yang Meng and Yuan Liu analysed the data and performed statistical analyses. Yuan Ma, Ziye Chen and Baoyi Liu wrote the manuscript. Tao Li, Chunqiao Liu, Runping Duan, Wen Ding, Kangjie Kong, Dianlei Guo and Jiali Ru critically reviewed and revised the manuscript.

## Funding

This work was supported by the National Natural Science Foundation of China (grant numbers 82271093 and 82070972 to Tao Li, and 32371015 to Chunqiao Liu), Guangdong Basic Research Center of Excellence for Major Blinding Eye Diseases Prevention and Treatment (2024‐RCPY‐009, 2024‐PIZC‐010) and the Research Funds of the State Key Laboratory of Ophthalmology (2025OZLHI09).

## Conflicts of Interest

The authors declare no conflicts of interest.

## Supporting information


**Figure S1:** Even distribution of microglia across the normoxic retinal vascular plexus and retinal planar regions at different postnatal ages.
**Figure S2:** Microglia variably increased throughout retinal layers and planar regions upon Oxygen‐induced retinopathy.
**Figure S3:** Experimental workflow, single‐cell RNA sequencing quality control, and cell type classification.
**Figure S4:** Fluorescence‐activated cell sorting (FACS) validates the efficacy of MACS‐enriched CD11b‐positive cells for scRNA‐seq and RT‐qPCR.
**Figure S5:** Time‐dependent reduction in BV2 viability upon hypoxia.
**Table S1:** Marker genes used for annotation of retinal cell clusters.
**Table S2:** Primer sets used for RT‐qPCR.

## Data Availability

The single‐cell RNA sequencing data have been uploaded at Gene Expression Omnibus (GEO) with accession number: GSE275394. All data supporting the findings of this study are provided in the text of this paper and related Supporting Information.
