# Supplementary material for: Stage‐Associated Microglial Subpopulations and Dynamics in Vascular Pathogenesis of Oxygen‐Induced Retinopathy
Source: Cell Prolif. 2026 Jan 22;59(7):e70165. doi: 10.1111/cpr.70165 (PMC13325556; doi:10.1111/cpr.70165)
Supplement: Supplementary file 1 — Figure S1: Even distribution of microglia across the normoxic retinal vascular plexus and retinal planar regions at different postnatal ages. Figure S2: Microglia variably increased throughout retinal layers and planar regions upon Oxygen‐induced retinopathy. Figure S3: Experimental workflow, single‐cell RNA sequencing quality control, and cell type classification. Figure S4: Fluorescence‐activated cell sorting (FACS) validates the efficacy of MACS‐enriched CD11b‐positive cells for scRNA‐seq and RT‐qPCR. Figure S5: Time‐dependent reduction in BV2 viability upon hypoxia. Table S1: Marker genes used for annotation of retinal cell clusters. Table S2: Primer sets used for RT‐qPCR. [file CPR-59-e70165-s001.docx]

**Supplementary Materials for**

**Stage-Associated Microglial Subpopulations and Dynamics in Vascular Pathogenesis of Oxygen-Induced Retinopathy**

***Authors:*** *Yuan Ma^1^*^†^*, Ziye Chen^1^*^†^*, Baoyi Liu^1^*^†^*, Wen Ding^1^, Runping Duan^1^, Kangjie Kong^1^, Zhuojun Xu^1^, Jizhu Li^1^, Jiali Ru^1^, Dianlei Guo^1^, Xiaoyue Wei^1^, Yaping Liu^1^, Zhuangling Lin^1^, Yang Meng^1^, Yuan Liu^1^, Lan Jiang^1^, Zitong Chen^1^, Rebiya Tuxun^1^, Chinling Tsai^1^, Chunqiao Liu^1^*, Tao Li^1^**

^1^ State Key Laboratory of Ophthalmology, Zhongshan Ophthalmic Center, Sun Yat-sen University, Guangdong Provincial Key Laboratory of Ophthalmology and Visual Science, Guangdong Provincial Clinical Research Center for Ocular Diseases; Guangzhou 510060, China.

† Yuan Ma, Ziye Chen and Baoyi Liu contributed equally to this work.

* To whom correspondence may be addressed.

Tao Li: [litao2@mail.sysu.edu.cn](mailto:litao2@mail.sysu.edu.cn) or Chunqiao Liu: liuchunq3@mail.sysu.edu.cn

**List of Supplementary Materials:**

**Supplementary Figure 1.** Even distribution of microglia across the normoxic retinal vascular plexus and retinal planar regions at different postnatal ages.

**Supplementary Figure 2.** Microglia variably increased throughout retinal layers and planar regions upon Oxygen-induced retinopathy.

**Supplementary Figure 3.** Experimental workflow, single-cell RNA sequencing quality control, and cell type classification.

**Supplementary Figure 4.** Fluorescence-activated cell sorting (FACS) validates the efficacy of MACS-enriched CD11b-positive cells for scRNA-seq and RT-qPCR.

**Supplementary Figure 5.** Time-dependent reduction in BV2 viability upon hypoxia.

**Supplementary Table 1.** Marker genes used for annotation of retinal cell clusters.

**Supplementary Table 2.** Primer sets used for RT-qPCR.


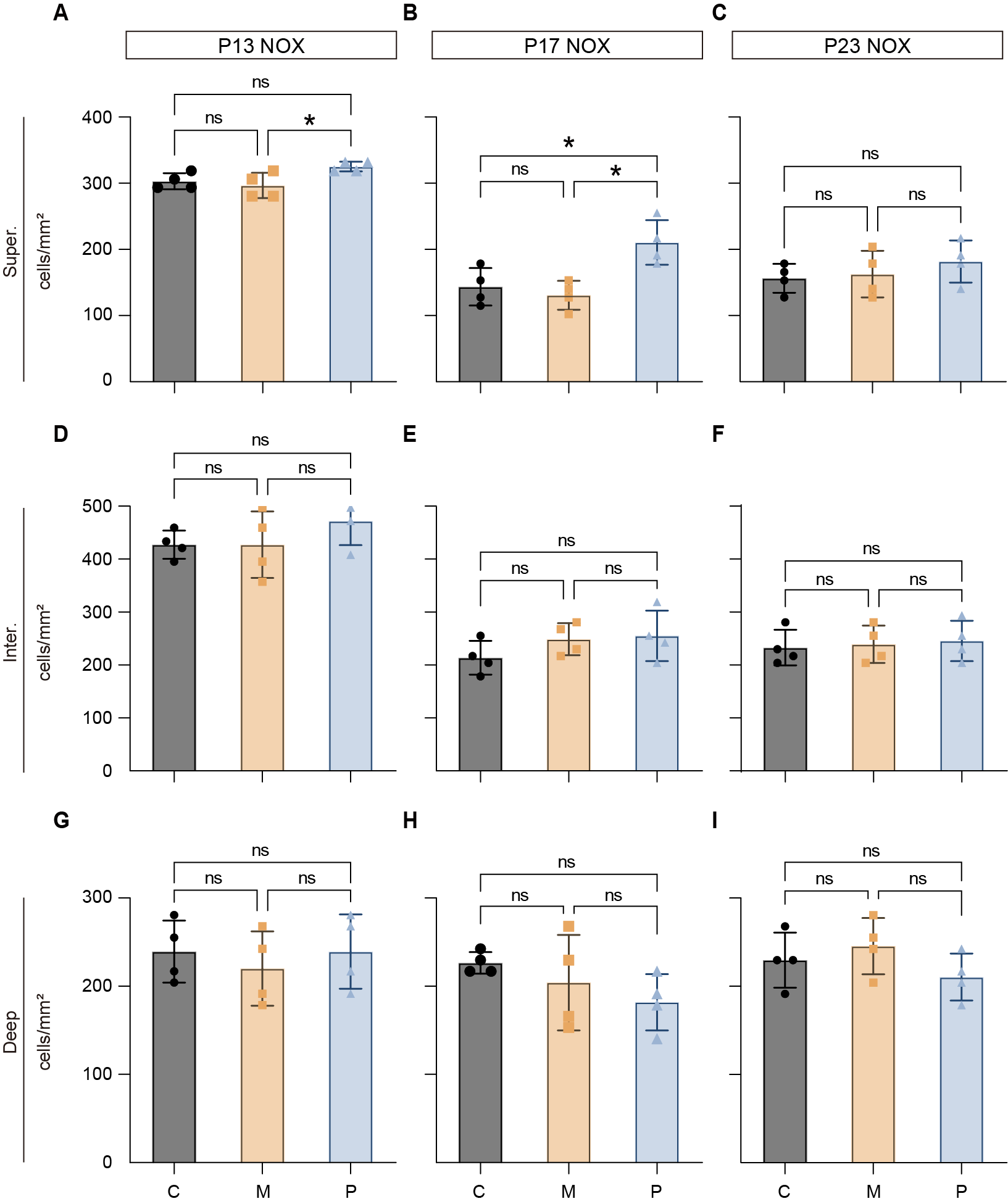


**Supplementary Figure 1.** **Even distribution of microglia across the normoxic retinal vascular plexus and retinal planar regions at different postnatal ages.** (**A-C**) Superficial layer.  (**D-F**) Intermediate layer. (**G-I**) Deep layer. (**A, D, G**) Postnatal day 13 (P13). (**B**, **E**, **H**) P17. (**C**, **F**, **I**) P23. Cell densities were compared between the central (C), midperipheral (M), and peripheral (P) regions. Data are presented as mean ± SD, n = 4 per group. Statistics were performed by a one-way ANOVA followed by Bonferroni test. ns, no significance; **P* < 0.05; ***P* < 0.01.


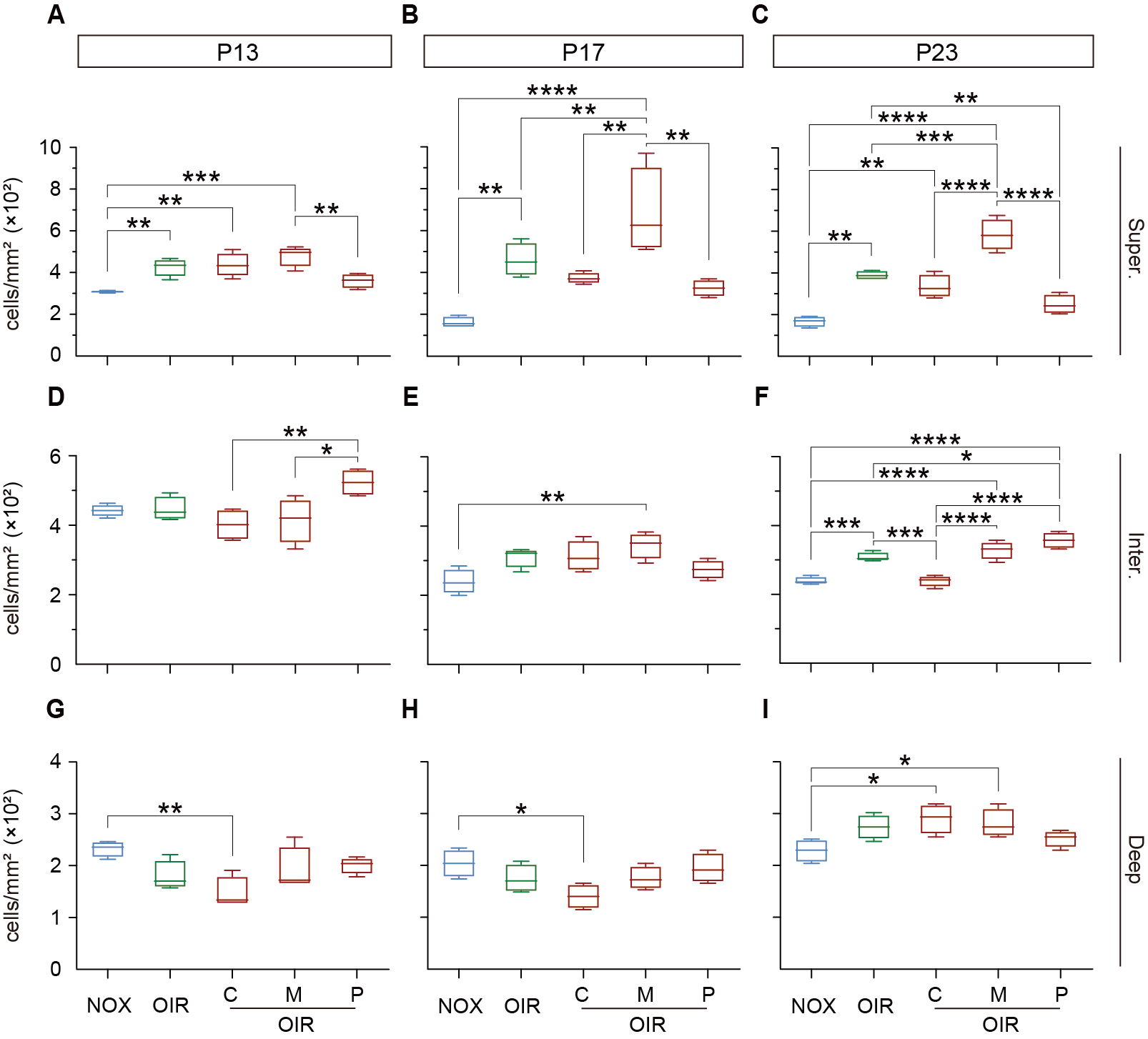


**Supplementary Figure 2.** **Microglia variably increased throughout retinal layers and planar regions upon Oxygen-induced retinopathy.** (**A-C**) Superficial layer. (**D-F**) Intermediate layer. (**G-I**) Deep layer. (**A, D, G**) Postnatal day 13 (P13). (**B**, **E**, **H**) P17. (**C**, **F**, **I**) P23. The first two boxes (NOX, blue; OIR, green) are average densities of microglia of different retinal layers from all retinal regions. Red boxes represent microglial densities in each OIR retinal layer from each region of central (C), midperipheral (M), and peripheral (P). n = 4 per group. Statistics were performed by one-way ANOVA followed by Bonferroni test. **P* < 0.05, ***P* < 0.01, ****P* < 0.001, *****P* < 0.0001.


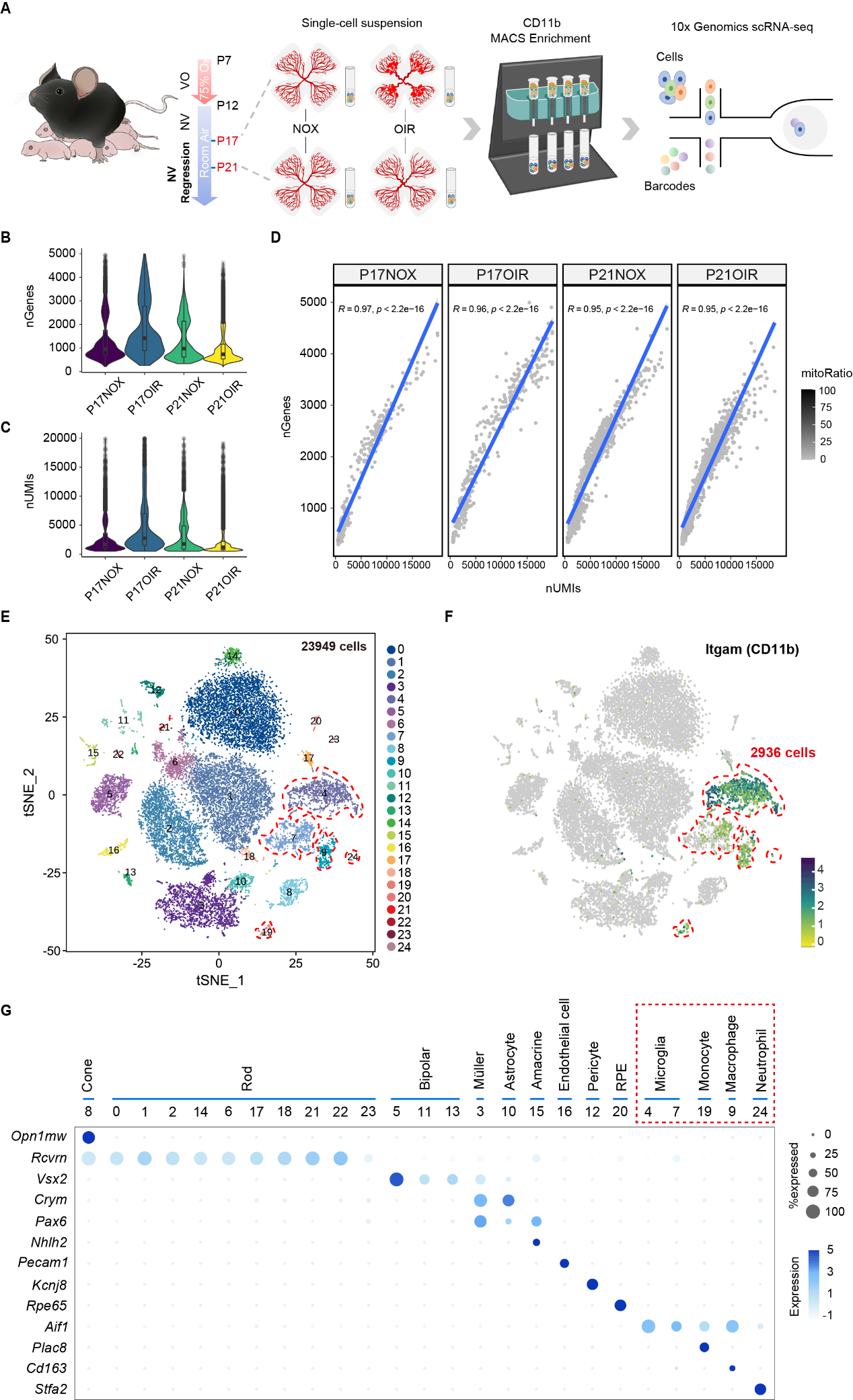


**Supplementary Figure 3.** **Experimental workflow, single-cell RNA sequencing quality control, and cell type classification.** (**A**) Schematic of the experimental design. Retinas were collected from mice on postnatal day 17 (P17) and P21 under either normoxic (NOX) or OIR condition. CD11b-positive cells were enriched from single-cell suspensions using Magnetic-Activated Cell Sorting (MACS) method. The enriched cells were then processed for 10X Genomics single-cell RNA sequencing. (**B, C**) Violin plots show the quality control metrics for the number of sequenced genes detected per cell (nGenes) (**B**) and the number of unique molecular identifiers (UMIs) caught per cell (nUMIs) (**C**) across the four samples (P17 NOX, P17 OIR, P21 NOX, P21 OIR). (**D**) Scatter plots reveal a strong positive correlation between nUMIs and nGenes from each sample. Grey scale represents percentage of mitochondrial genes (mitoRatio) out of total sequenced transcripts with low levels indicating high-quality of the dataset. Pearson correlation coefficient (R) and *p*-value are shown for each plot. (**E**) t-Distributed Stochastic Neighbor Embedding (t-SNE) visualization of all 23,949 cells after data integration and clustering. The analysis identified 25 distinct cell clusters (labeled 0-24). Red dashed circles demarcate clusters 4, 7, 9, 19, and 24, which correspond to CD11b positive myeloid cell populations. (**F**) CD11b expression in myeloid clusters containing 2,936 cells on t-SNE plot. (**G**) Expression of canonical marker genes used to assign cell clusters. The dot size represents the percentage of cells in a cluster expressing the gene (% expressed), and the color intensity represents the average expression level (expression). The identified major cell types include photoreceptors (Rods, Cones), neurons (Bipolar), glia (Müller, Astrocyte), vascular cells (Endothelial cell, Pericyte), and immune cells (Microglia, Monocyte, Macrophage, Neutrophil). The red box highlights the myeloid cell clusters in (**E**) and (**F**).

**
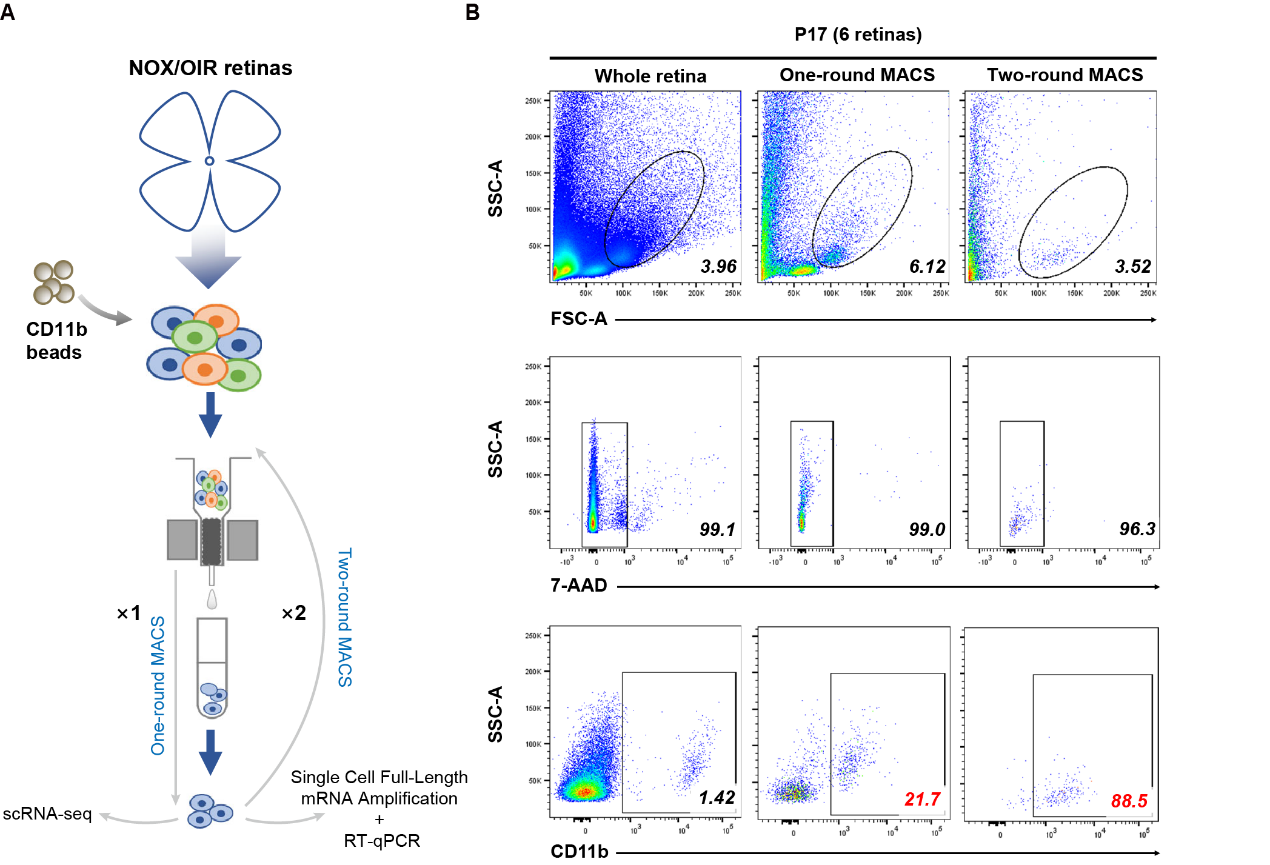
**

**Supplementary Figure 4.** **Fluorescence-activated cell sorting (FACS) validates the efficacy of MACS-enriched CD11b-positive cells for scRNA-seq and RT-qPCR.** (**A**) Schematic of the experimental workflow. Isolated cells were enriched through MACS using CD11b microbeads. Cells underwent one-round enrichment were used for scRNA-seq, while two-round enrichment were for RT-qPCR. (**B**) Flow cytometry analysis inspection of the purity of enriched cells. Representative plots show the proportion of CD11b-positive cells from a pool of 6 retinas from P17 mice without sorting (Whole retina), one-round enrichment, and two-round enrichment.

**
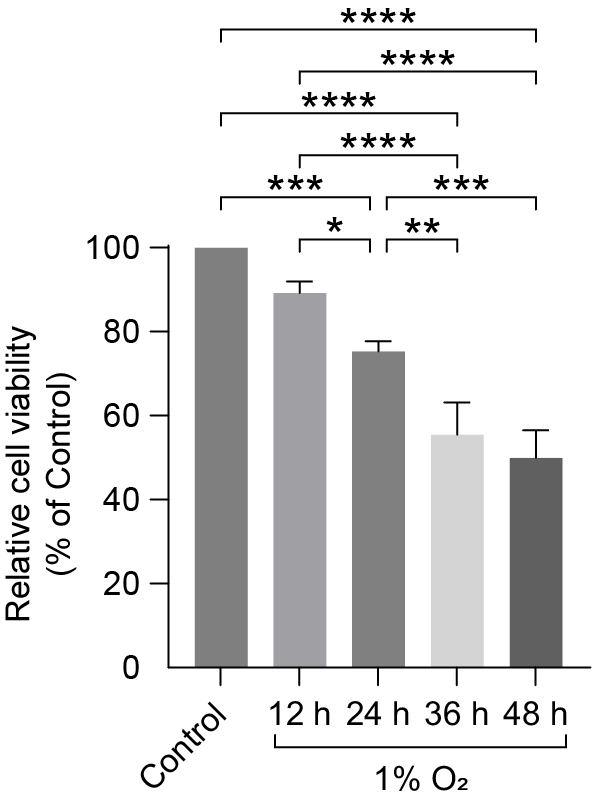
**

**Supplementary Figure 5. Time-dependent reduction in BV2 viability upon hypoxia.**  BV2 cells were cultured under normoxia (Control) or exposed to hypoxic conditions (1% O₂) for 12, 24, 36, and 48 h. Cell viability was assessed using the CCK-8 assay. Data was normalized to the control and presented as percentage. Note the viability reduced over hypoxic time. All data are presented as the mean ± SD. n = 3. Statistical significance was determined by one-way ANOVA followed by Bonferroni test. **P* < 0.05, ***P* < 0.01, ****P* < 0.001, *****P* < 0.0001.

**Supplementary Table 1. Marker genes used for annotation of retinal cell clusters.**

|  | |
| --- | --- |
| **MARKERS_PHOTORECEPTOR_RODS** | |
| **MARKER** | **REFERENCE** |
| *Rho* | Qiang et al. (2018) (DOI: 10.1016/j.jphotobiol.2018.04.003); Kaewkhaw et al. (2015) (DOI: 10.1002/stem.2122) |
| *Nrl* | Kaewkhaw et al. (2015) (DOI: 10.1002/stem.2122); Sanes & Zipursky (2010) (DOI: 10.1016/j.neuron.2010.01.018) |
| *Nr2e3* | Kaewkhaw et al. (2015) (DOI: 10.1002/stem.2122); Sanes & Zipursky (2010) (DOI: 10.1016/j.neuron.2010.01.018) |
| *Gnat1* | Kaewkhaw et al. (2015) (DOI: 10.1002/stem.2122); Sanes & Zipursky (2010) (DOI: 10.1016/j.neuron.2010.01.018) |
| *Cngb1* | Kaewkhaw et al. (2015) (DOI: 10.1002/stem.2122) |
| *Essrb* | Kaewkhaw et al. (2015) (DOI: 10.1002/stem.2122) |
| *Mef2c* | Kaewkhaw et al. (2015) (DOI: 10.1002/stem.2122) |
| *Gabrr2* | Kaewkhaw et al. (2015) (DOI: 10.1002/stem.2122) |
| *Cplx4* | Pinelli et al. (2016) (DOI: 10.1093/nar/gkw486) |
| *Cnga1* | Macosko et al. (2015) (DOI: 10.1016/j.cell.2015.05.002); Postel et al. (2013) (http://www.molvis.org/molvis/v19/2058) |
| *Nxnl1* | Ait-Ali et al. (2015) (DOI: 10.1016/j.cell.2015.03.023) |
|  |  |
| **MARKERS_PHOTORECEPTOR_CONES** | |
| **MARKER** | **REFERENCE** |
| *Opn1mw* | Kaewkhaw et al. (2015) (DOI: 10.1002/stem.2122); Ait-Ali et al. (2015) (DOI: 10.1016/j.cell.2015.03.023) |
| *Opn1sw* | Kaewkhaw et al. (2015) (DOI: 10.1002/stem.2122); Ait-Ali et al. (2015) (DOI: 10.1016/j.cell.2015.03.023) |
| *Arr3* | Kaewkhaw et al. (2015) (DOI: 10.1002/stem.2122); Ait-Ali et al. (2015) (DOI: 10.1016/j.cell.2015.03.023) |
| *Gnat2* | Macosko et al. (2015) (DOI: 10.1016/j.cell.2015.05.002); Welby et al. (2017) (DOI: 10.1016/j.stemcr.2017.10.018) |
| *Cabp5* | Shekhar et al. (2016) (DOI: 10.1016/j.cell.2016.07.054) |
| *Rxrg* | Kaewkhaw et al. (2015) (DOI: 10.1002/stem.2122) |
| *Thrb* | Welby et al. (2017) (DOI: 10.1016/j.stemcr.2017.10.018) |
|  |  |
| **MARKERS_BIPOLAR CELLS** | |
| **MARKER** | **REFERENCE** |
| *Cabp5* | Haeseleer et al. (2000) (DOI: 10.1074/jbc.275.2.1247); Ueda et al. (1997) (DOI: 10.1523/JNEUROSCI.17-09-03014.1997) |
| *Grm6* | Masu et al. (1995) (https://www.ncbi.nlm.nih.gov/pubmed/7889569); |
| *Isl1* | Welby et al. (2017) (DOI: 10.1016/j.stemcr.2017.10.018) |
| *Otx2* | Baas et al. (2000) (DOI: 10.1016/S0169-328X(00)00060-7) |
| *Vsx2* | Burmeister et al. (1996) (DOI: 10.1038/ng0496-376) |
| *Slc1a2* | Welby et al. (2017) (DOI: 10.1016/j.stemcr.2017.10.018) |
| *Lhx3* | Welby et al. (2017) (DOI: 10.1016/j.stemcr.2017.10.018) |
|  |  |
| **MARKERS_AMACRINE CELLS** | |
| **MARKER** | **REFERENCE** |
| *Prox1* | Kunzevitzky et al. (2010) (DOI: 10.1167/iovs.09-4540); Macosko et al. (2015) (DOI: 10.1016/j.cell.2015.05.002); Voinescu et al. (2009) (DOI: 10.1002/cne.22200) |
| *Pax6* | Kunzevitzky et al. (2010) (DOI: 10.1167/iovs.09-4540); Macosko et al. (2015) (DOI: 10.1016/j.cell.2015.05.002); Voinescu et al. (2009) (DOI: 10.1002/cne.22200) |
| *Stx1a* | Kunzevitzky et al. (2010) (DOI: 10.1167/iovs.09-4540); Macosko et al. (2015) (DOI: 10.1016/j.cell.2015.05.002); Voinescu et al. (2009) (DOI: 10.1002/cne.22200) |
| *Gad2* | Kunzevitzky et al. (2010) (DOI: 10.1167/iovs.09-4540); Macosko et al. (2015) (DOI: 10.1016/j.cell.2015.05.002); Voinescu et al. (2009) (DOI: 10.1002/cne.22200) |
| *Slc6a9* | Kunzevitzky et al. (2010) (DOI: 10.1167/iovs.09-4540); Macosko et al. (2015) (DOI: 10.1016/j.cell.2015.05.002); Voinescu et al. (2009) (DOI: 10.1002/cne.22200) |
| *Npy* | Kunzevitzky et al. (2010) (DOI: 10.1167/iovs.09-4540); Macosko et al. (2015) (DOI: 10.1016/j.cell.2015.05.002); Voinescu et al. (2009) (DOI: 10.1002/cne.22200) |
| *Ebf1* | Kunzevitzky et al. (2010) (DOI: 10.1167/iovs.09-4540); Macosko et al. (2015) (DOI: 10.1016/j.cell.2015.05.002); Voinescu et al. (2009) (DOI: 10.1002/cne.22200) |
| *Nrxn2* | Kunzevitzky et al. (2010) (DOI: 10.1167/iovs.09-4540); Macosko et al. (2015) (DOI: 10.1016/j.cell.2015.05.002); Voinescu et al. (2009) (DOI: 10.1002/cne.22200) |
| *Calb1* | Kunzevitzky et al. (2010) (DOI: 10.1167/iovs.09-4540); Macosko et al. (2015) (DOI: 10.1016/j.cell.2015.05.002); Voinescu et al. (2009) (DOI: 10.1002/cne.22200) |
| *Calr* | Kunzevitzky et al. (2010) (DOI: 10.1167/iovs.09-4540); Macosko et al. (2015) (DOI: 10.1016/j.cell.2015.05.002); Voinescu et al. (2009) (DOI: 10.1002/cne.22200) |
| *Pvalb* | Kunzevitzky et al. (2010) (DOI: 10.1167/iovs.09-4540); Macosko et al. (2015) (DOI: 10.1016/j.cell.2015.05.002); Voinescu et al. (2009) (DOI: 10.1002/cne.22200) |
| *Lrrn3* | Kunzevitzky et al. (2010) (DOI: 10.1167/iovs.09-4540) |
| *Tfap2b* | Kunzevitzky et al. (2010) (DOI: 10.1167/iovs.09-4540) |
| *Mark2* | Kunzevitzky et al. (2010) (DOI: 10.1167/iovs.09-4540) |
| *Kif2c* | Kunzevitzky et al. (2010) (DOI: 10.1167/iovs.09-4540) |
| *Lims1* | Kunzevitzky et al. (2010) (DOI: 10.1167/iovs.09-4540) |
| *Fzd3* | Kunzevitzky et al. (2010) (DOI: 10.1167/iovs.09-4540) |
|  |  |
| **MARKERS_HORIZONTAL CELLS** | |
| **MARKER** | **REFERENCE** |
| *Lhx1* | Macosko et al. (2015) (DOI: 10.1016/j.cell.2015.05.002) |
| *Pax6* | Macosko et al. (2015) (DOI: 10.1016/j.cell.2015.05.002) |
| *Calb2* | Voinescu et al. (2009) (DOI: 10.1002/cne.22200) |
| *Prox1* | de Melo et al. (2011) (DOI: 10.1242/dev.061846) |
|  |  |
| **MARKERS_RETINAL GANGLION CELLS** | |
| **MARKER** | **REFERENCE** |
| *Rbpms* | Rodriguez et al. (2014) (DOI: 10.1002/cne.23521) |
| *Opn4* | Rodriguez et al. (2014) (DOI: 10.1002/cne.23521) |
| *Rbfox3* | Rodriguez et al. (2014) (DOI: 10.1002/cne.23521) |
| *Tubb3* | Jiang et al. (2016) (DOI: 10.1111/jnc.13400); Struebing et al. (2016) (DOI: 10.3389/fgene.2016.00169) |
| *Thy1* | Struebing et al. (2016) (DOI: 10.3389/fgene.2016.00169); Pegu et al. (2015) (DOI: 10.1038/ncomms9447) |
| *Nrn1* | Sharma et al. (2015) (DOI: 10.1038/cddis.2015.22); Ivanov et al. (2006) (DOI: 10.1016/j.febslet.2005.12.017) |
| *Nrg1* | Ivanov et al. (2006) (DOI: 10.1016/j.febslet.2005.12.017); Yang et al. (2015) (DOI: 10.15252/emmm.201404922) |
|  |  |
| **MARKERS_MULLER CELLS** | |
| **MARKER** | **REFERENCE** |
| *Gfap* | Roesch et al. (2008) (DOI: 10.1002/cne.21730) |
| *Vim* | Macosko et al. (2015) (DOI: 10.1016/j.cell.2015.05.002) |
| *Aqp4* | Macosko et al. (2015) (DOI: 10.1016/j.cell.2015.05.002) |
| *S100B* | Roesch et al. (2008) (DOI: 10.1002/cne.21730) |
| *Aldh1l1* | Cahoy et al. (2008) (DOI: 10.1523/JNEUROSCI.4178-07.2008) |
| *Glul* | Macosko et al. (2015) (DOI: 10.1016/j.cell.2015.05.002); Roesch et al. (2008) (DOI: 10.1002/cne.21730) |
| *Rlbp1* | Macosko et al. (2015) (DOI: 10.1016/j.cell.2015.05.002); Roesch et al. (2008) (DOI: 10.1002/cne.21730) |
| *Slc1a3* | Macosko et al. (2015) (DOI: 10.1016/j.cell.2015.05.002) |
| *Pax6* | Macosko et al. (2015) (DOI: 10.1016/j.cell.2015.05.002) |
| *Acsl3* | Macosko et al. (2015) (DOI: 10.1016/j.cell.2015.05.002) |
| *Sparc* | Macosko et al. (2015) (DOI: 10.1016/j.cell.2015.05.002) |
| *Col9a1* | Macosko et al. (2015) (DOI: 10.1016/j.cell.2015.05.002) |
| *Spc25* | Macosko et al. (2015) (DOI: 10.1016/j.cell.2015.05.002) |
| *Car14* | Macosko et al. (2015) (DOI: 10.1016/j.cell.2015.05.002) |
| *Apoe* | Macosko et al. (2015) (DOI: 10.1016/j.cell.2015.05.002); Roesch et al. (2008) (DOI: 10.1002/cne.21730) |
| *Dkk3* | Macosko et al. (2015) (DOI: 10.1016/j.cell.2015.05.002); Roesch et al. (2008) (DOI: 10.1002/cne.21730) |
| *Itm2B* | Roesch et al. (2008) (DOI: 10.1002/cne.21730) |
| *Dbi* | Macosko et al. (2015) (DOI: 10.1016/j.cell.2015.05.002); Roesch et al. (2008) (DOI: 10.1002/cne.21730) |
| *Gpr37* | Macosko et al. (2015) (DOI: 10.1016/j.cell.2015.05.002); Roesch et al. (2008) (DOI: 10.1002/cne.21730) |
| *Car2* | Macosko et al. (2015) (DOI: 10.1016/j.cell.2015.05.002); Roesch et al. (2008) (DOI: 10.1002/cne.21730) |
| *Dtx2* | Roesch et al. (2008) (DOI: 10.1002/cne.21730) |
| *Pax6* | Macosko et al. (2015) (DOI: 10.1016/j.cell.2015.05.002); Roesch et al. (2008) (DOI: 10.1002/cne.21730) |
| *Clu* | Macosko et al. (2015) (DOI: 10.1016/j.cell.2015.05.002); Roesch et al. (2008) (DOI: 10.1002/cne.21730) |
| *Ptn* | Macosko et al. (2015) (DOI: 10.1016/j.cell.2015.05.002); Roesch et al. (2008) (DOI: 10.1002/cne.21730) |
| *Pdgfra* | Roesch et al. (2008) (DOI: 10.1002/cne.21730) |
| *Hes1* | Ueno et al. (2017) (DOI: 10.1038/s41598-017-03874-8) |
|  |  |
| **MARKERS_ASTROCYTES** | |
| **MARKER** | **REFERENCE** |
| *Gfap* | Macosko et al. (2015) (DOI: 10.1016/j.cell.2015.05.002); Liddelow et al. (2017) (DOI: 10.1038/nature21029) |
| *Vim* | Macosko et al. (2015) (DOI: 10.1016/j.cell.2015.05.002); Liddelow et al. (2017) (DOI: 10.1038/nature21029) |
| *Aqp4* | Clarke et al. (2018) (DOI: 10.1073/pnas.1800165115) |
| *S100B* | Macosko et al. (2015) (DOI: 10.1016/j.cell.2015.05.002) |
| *Aldh1l1* | Cahoy et al. (2008) (DOI: 10.1523/JNEUROSCI.4178-07.2008) |
| *Slc1a2* | Cahoy et al. (2008) (DOI: 10.1523/JNEUROSCI.4178-07.2008) |
| *Slc1a3* | Cahoy et al. (2008) (DOI: 10.1523/JNEUROSCI.4178-07.2008) |
| *Fgfr3* | Cahoy et al. (2008) (DOI: 10.1523/JNEUROSCI.4178-07.2008) |
| *Rlbp1* | Macosko et al. (2015) (DOI: 10.1016/j.cell.2015.05.002) |
| *Dbi* | Macosko et al. (2015) (DOI: 10.1016/j.cell.2015.05.002) |
| *Apoe* | Macosko et al. (2015) (DOI: 10.1016/j.cell.2015.05.002) |
| *Glul* | Macosko et al. (2015) (DOI: 10.1016/j.cell.2015.05.002) |
| *Pak3* | Macosko et al. (2015) (DOI: 10.1016/j.cell.2015.05.002) |
| *Clu* | Macosko et al. (2015) (DOI: 10.1016/j.cell.2015.05.002) |
| *Ptn* | Macosko et al. (2015) (DOI: 10.1016/j.cell.2015.05.002) |
| *Pdgfra* | Macosko et al. (2015) (DOI: 10.1016/j.cell.2015.05.002) |
| *Gjb6* | Cahoy et al. (2008) (DOI: 10.1523/JNEUROSCI.4178-07.2008) |
| *Aldoc* | Macosko et al. (2015) (DOI: 10.1016/j.cell.2015.05.002); Cahoy et al. (2008) (DOI: 10.1523/JNEUROSCI.4178-07.2008) |
| *Ntsr2* | Cahoy et al. (2008) (DOI: 10.1523/JNEUROSCI.4178-07.2008) |
| *Acsbg1* | Cahoy et al. (2008) (DOI: 10.1523/JNEUROSCI.4178-07.2008) |
| *Rfx4* | Cahoy et al. (2008) (DOI: 10.1523/JNEUROSCI.4178-07.2008) |
| *Pbxip1* | Cahoy et al. (2008) (DOI: 10.1523/JNEUROSCI.4178-07.2008) |
| *Gli3* | Cahoy et al. (2008) (DOI: 10.1523/JNEUROSCI.4178-07.2008) |
| *Gja1* | Macosko et al. (2015) (DOI: 10.1016/j.cell.2015.05.002) |
| *Timp1* | Liddelow et al. (2017) (DOI: 10.1038/nature21029) |
| *Aspg* | Liddelow et al. (2017) (DOI: 10.1038/nature21029) |
| *Lcn2* | Liddelow et al. (2017) (DOI: 10.1038/nature21029) |
| *Steap4* | Liddelow et al. (2017) (DOI: 10.1038/nature21029) |
| *S1pr3* | Liddelow et al. (2017) (DOI: 10.1038/nature21029) |
| *Hspb1* | Liddelow et al. (2017) (DOI: 10.1038/nature21029) |
| *Cxcl10* | Liddelow et al. (2017) (DOI: 10.1038/nature21029) |
| *Cd44* | Liddelow et al. (2017) (DOI: 10.1038/nature21029) |
| *Osmr* | Liddelow et al. (2017) (DOI: 10.1038/nature21029) |
| *Serpina3n* | Liddelow et al. (2017) (DOI: 10.1038/nature21029) |
|  |  |
| **MARKERS_IMMUNE CELLS** | |
| **MARKER** | **REFERENCE** |
| **Microglia** | **Main focus** |
| *Cx3cr1* | Sousa et al. (2017) (DOI: 10.3389/fimmu.2017.00198); Keren-Shaul et al. (2017) (DOI: 10.1016/j.cell.2017.05.018); Olah et al. (2018) (DOI: 10.1038/s41467-018-02926-5); Skelly et al. (2018) (DOI: 10.1016/j.celrep.2017.12.072) |
| *P2ry12* | Sousa et al. (2017) (DOI: 10.3389/fimmu.2017.00198); Olah et al. (2018) (DOI: 10.1038/s41467-018-02926-5); Greter et al. (2015) (DOI: 10.3389/fimmu.2015.00249); Wes et al. (2016) (DOI: 10.1002/glia.22866) |
| *Siglech* | Mrdjen et al. (2018) (DOI: 10.1016/j.immuni.2018.01.011); Sousa et al. (2017) (DOI: 10.3389/fimmu.2017.00198); Skelly et al. (2018) (DOI: 10.1016/j.celrep.2017.12.072); Chiu et al. (2013) (DOI: 10.1016/j.celrep.2013.06.018); Greter et al. (2015) (DOI: 10.3389/fimmu.2015.00249) |
| *Hexb* | Sousa et al. (2017) (DOI: 10.3389/fimmu.2017.00198); Keren-Shaul et al. (2017) (DOI: 10.1016/j.cell.2017.05.018); Wes et al. (2016) (DOI: 10.1002/glia.22866) |
| *Tmem119* | Sousa et al. (2017) (DOI: 10.3389/fimmu.2017.00198); Chiu et al. (2013) (DOI: 10.1016/j.celrep.2013.06.018); Wes et al. (2016) (DOI: 10.1002/glia.22866) |
| *Trem2* | Sousa et al. (2017) (DOI: 10.3389/fimmu.2017.00198); Keren-Shaul et al. (2017) (DOI: 10.1016/j.cell.2017.05.018); Olah et al. (2018) (DOI: 10.1038/s41467-018-02926-5) |
| *Csf1r* | Clara Beutner et al. (2013) (DOI: 10.1002/glia.22524) |
| *Fcrl* | Timothy R Hammond et al (2018) (DOI: 10.1016/j.immuni.2018.11.004) |
| *Sparc* | Timothy R Hammond et al (2018) (DOI: 10.1016/j.immuni.2018.11.004) |
| *C1qa* | Clara Beutner et al. (2013) (DOI: 10.1002/glia.22524) |
| *Aif1* | Clara Beutner et al. (2013) (DOI: 10.1002/glia.22524) |
| *Itgam* | Clara Beutner et al. (2013) (DOI: 10.1002/glia.22524) |
| *Dock2* | Clara Beutner et al. (2013) (DOI: 10.1002/glia.22524) |
| *Tmsb4x* | Chang He et al. (2021) (DOI: 10.1073/pnas.2023290118) |
| *P2rx7* | Clara Beutner et al. (2013) (DOI: 10.1002/glia.22524) |
| *Olfml3* | Sousa et al. (2017) (DOI: 10.3389/fimmu.2017.00198); Chiu et al. (2013) (DOI: 10.1016/j.celrep.2013.06.018); Wes et al. (2016) (DOI: 10.1002/glia.22866) |
| *Crybb1* | Sousa et al. (2017) (DOI: 10.3389/fimmu.2017.00198); Wes et al. (2016) (DOI: 10.1002/glia.22866) |
| *Adora3* | Sousa et al. (2017) (DOI: 10.3389/fimmu.2017.00198) |
| *Bcl2a1a* | Sousa et al. (2017) (DOI: 10.3389/fimmu.2017.00198) |
| *Mertk* | Mrdjen et al. (2018) (DOI: 10.1016/j.immuni.2018.01.011) |
| *Tyrobp* | Sousa et al. (2017) (DOI: 10.3389/fimmu.2017.00198); Keren-Shaul et al. (2017) (DOI: 10.1016/j.cell.2017.05.018) |
| *P2ry13* | Sousa et al. (2017) (DOI: 10.3389/fimmu.2017.00198); Wes et al. (2016) (DOI: 10.1002/glia.22866) |
| *Gpr34* | Olah et al. (2018) (DOI: 10.1038/s41467-018-02926-5); Skelly et al. (2018) (DOI: 10.1016/j.celrep.2017.12.072); Wes et al. (2016) (DOI: 10.1002/glia.22866) |
| *Cd74* | Olah et al. (2018) (DOI: 10.1038/s41467-018-02926-5) |
| **Inflammatory macrophages** | **Distinguishing** |
| *F13a1* | Timothy R Hammond et al (2018) (DOI: 10.1016/j.immuni.2018.11.004) |
| *Lyve1* | Timothy R Hammond et al (2018) (DOI: 10.1016/j.immuni.2018.11.005) |
| *Mgl2* | Timothy R Hammond et al (2018) (DOI: 10.1016/j.immuni.2018.11.006) |
| *Emr1(F4/80)* | Timothy R Hammond et al (2018) (DOI: 10.1016/j.immuni.2018.11.007) |
| *Tfrc(CD71)* | Timothy R Hammond et al (2018) (DOI: 10.1016/j.immuni.2018.11.008) |
| *Csf1r(CD115)* | Timothy R Hammond et al (2018) (DOI: 10.1016/j.immuni.2018.11.009) |
| *Sirpa(CD172a)* | Timothy R Hammond et al (2018) (DOI: 10.1016/j.immuni.2018.11.010) |
| *Nramp1* | Timothy R Hammond et al (2018) (DOI: 10.1016/j.immuni.2018.11.011) |
| *Mrc1* | Timothy R Hammond et al (2018) (DOI: 10.1016/j.immuni.2018.11.012) |
| *Cd163* | Timothy R Hammond et al (2018) (DOI: 10.1016/j.immuni.2018.11.013) |
| *H2-Aa* | Ronning et al. (2019) (DOI: 10.1038/s41598-019-41141-0) |
| *Cd74* | Ronning et al. (2019) (DOI: 10.1038/s41598-019-41141-0); Nirmal et al. (2018) (DOI: 10.1158/2326-6066.CIR-18-0342) |
| *H2-Ab1* | Ronning et al. (2019) (DOI: 10.1038/s41598-019-41141-0) |
| *H2-Eb1* | Ronning et al. (2019) (DOI: 10.1038/s41598-019-41141-0) |
| *H2-DMb1* | Ronning et al. (2019) (DOI: 10.1038/s41598-019-41141-0) |
| *Il1b* | Ronning et al. (2019) (DOI: 10.1038/s41598-019-41141-0) |
| *Tgfbi* | Ronning et al. (2019) (DOI: 10.1038/s41598-019-41141-0) |
| *Ctsc* | Ronning et al. (2019) (DOI: 10.1038/s41598-019-41141-0) |
| **Monocytes** | **Distinguishing** |
| *Acp5* | Ronning et al. (2019) (DOI: 10.1038/s41598-019-41141-0) |
| *Lyz2* | Ronning et al. (2019) (DOI: 10.1038/s41598-019-41141-0) |
| *Lgals3* | Ronning et al. (2019) (DOI: 10.1038/s41598-019-41141-0) |
| *Ms4a7* | Ronning et al. (2019) (DOI: 10.1038/s41598-019-41141-0) |
| *Apoe* | Ronning et al. (2019) (DOI: 10.1038/s41598-019-41141-0) |
| *Ccr2* | Timothy R Hammond et al (2018) (DOI: 10.1016/j.immuni.2018.11.009) |
| *Cfp* | Timothy R Hammond et al (2018) (DOI: 10.1016/j.immuni.2018.11.010) |
| *Ly6c2* | Timothy R Hammond et al (2018) (DOI: 10.1016/j.immuni.2018.11.011) |
| *Plac8* | Timothy R Hammond et al (2018) (DOI: 10.1016/j.immuni.2018.11.012) |
| **Neutrophils** | **Distinguishing** |
| *Sod2* | François Binet et al. (2020) ( DOI: 10.1126/science.aay5356) |
| *S100a8* | François Binet et al. (2020) ( DOI: 10.1126/science.aay5357) |
| *Tlr4* | François Binet et al. (2020) ( DOI: 10.1126/science.aay5358) |
| *Plaur* | François Binet et al. (2020) ( DOI: 10.1126/science.aay5359) |
| **T cells, Natural killer cells** | **Distinguishing** |
| *Cd3g* | Zhong et al. (2017) (DOI: 10.1038/s41598-017-18195-z); Nirmal et al. (2018) (DOI: 10.1158/2326-6066.CIR-18-0342); Skelly et al. (2018) (DOI: 10.1016/j.celrep.2017.12.072) |
| *Cd3d* | Zhong et al. (2017) (DOI: 10.1038/s41598-017-18195-z); Nirmal et al. (2018) (DOI: 10.1158/2326-6066.CIR-18-0342); Skelly et al. (2018) (DOI: 10.1016/j.celrep.2017.12.072) |
| *Cd3e* | Zhong et al. (2017) (DOI: 10.1038/s41598-017-18195-z); Nirmal et al. (2018) (DOI: 10.1158/2326-6066.CIR-18-0342); Skelly et al. (2018) (DOI: 10.1016/j.celrep.2017.12.072) |
| *Nkg7* | Keren-Shaul et al. (2017) (DOI: 10.1016/j.cell.2017.05.018); Skelly et al. (2018) (DOI: 10.1016/j.celrep.2017.12.072) |
| *Ncr1* | Skelly et al. (2018) (DOI: 10.1016/j.celrep.2017.12.072); Zhong et al. (2017) (DOI: 10.1038/s41598-017-18195-z); Bezman et al. (2012) (DOI: 10.1038/ni.2395) |
| *Klrd1* | Zhong et al. (2017) (DOI: 10.1038/s41598-017-18195-z); Nirmal et al. (2018) (DOI: 10.1158/2326-6066.CIR-18-0342); Skelly et al. (2018) (DOI: 10.1016/j.celrep.2017.12.072) |
| *Klrc2* | Bezman et al. (2012) (DOI: 10.1038/ni.2395); Nirmal et al. (2018) (DOI: 10.1158/2326-6066.CIR-18-0342) |
| *Klrk1* | Bezman et al. (2012) (DOI: 10.1038/ni.2395); Skelly et al. (2018) (DOI: 10.1016/j.celrep.2017.12.072) |
|  | |
| **MARKERS_ ENDOTHELIAL CELLS** | |
| **MARKER** | **REFERENCE** |
| *Vwf* | Yakimchuk (2013) (DOI: 10.13070/mm.en.3.183) |
| *Cdh5* | Yakimchuk (2013) (DOI: 10.13070/mm.en.3.183) |
| *Tek* | Sato et al. (1995) DOI: 10.1038/376070a0 |
| *Pecam1* | Yakimchuk (2013) (DOI: 10.13070/mm.en.3.183) |
| *Flt1* | Yakimchuk (2013) (DOI: 10.13070/mm.en.3.183) |
| *Kdr* | Yakimchuk (2013) (DOI: 10.13070/mm.en.3.183) |
| *Nos3* | Yakimchuk (2013) (DOI: 10.13070/mm.en.3.183) |
| *Mcam* | Yakimchuk (2013) (DOI: 10.13070/mm.en.3.183) |
| *Mmrn1* | Bhasin et al. (2010) (DOI: 10.1186/1471-2164-11-342) |
| *Cldn5* | Bhasin et al. (2010) (DOI: 10.1186/1471-2164-11-342) |
| *Bmx* | Bhasin et al. (2010) (DOI: 10.1186/1471-2164-11-342) |
| *Angpt2* | Bhasin et al. (2010) (DOI: 10.1186/1471-2164-11-342) |
| *Gja4* | Bhasin et al. (2010) (DOI: 10.1186/1471-2164-11-342) |
| *Tie1* | Bhasin et al. (2010) (DOI: 10.1186/1471-2164-11-342) |
| *Robo4* | Bhasin et al. (2010) (DOI: 10.1186/1471-2164-11-342) |
| *Ecscr* | Bhasin et al. (2010) (DOI: 10.1186/1471-2164-11-342) |
|  |  |
| **Markers_pericytes** | |
| **Marker** | **REFERENCE** |
| *Pdgfrb* | Armulik et al. (2011) (DOI: 10.1016/j.devcel.2011.07.001) |
| *Des* | Armulik et al. (2011) (DOI: 10.1016/j.devcel.2011.07.001) |
| *Cspg4* | Armulik et al. (2011) (DOI: 10.1016/j.devcel.2011.07.001) |
| *Acta2* | Armulik et al. (2011) (DOI: 10.1016/j.devcel.2011.07.001) |
| *Anpep* | Armulik et al. (2011) (DOI: 10.1016/j.devcel.2011.07.001) |
| *Rgs5* | Armulik et al. (2011) (DOI: 10.1016/j.devcel.2011.07.001) |
| *Abcc9* | Armulik et al. (2011) (DOI: 10.1016/j.devcel.2011.07.001) |
| *Kcnj8* | Armulik et al. (2011) (DOI: 10.1016/j.devcel.2011.07.001) |
| *Cd248* | Armulik et al. (2011) (DOI: 10.1016/j.devcel.2011.07.001) |
| *Dlk1* | Armulik et al. (2011) (DOI: 10.1016/j.devcel.2011.07.001) |
| *Mcam* | Armulik et al. (2011) (DOI: 10.1016/j.devcel.2011.07.001) |
| *Nt5e* | Armulik et al. (2011) (DOI: 10.1016/j.devcel.2011.07.001) |
| *Cd44* | Armulik et al. (2011) (DOI: 10.1016/j.devcel.2011.07.001) |
| *Angpt1* | Armulik et al. (2011) (DOI: 10.1016/j.devcel.2011.07.001) |
|  |  |
| **MARKERS_FIBROBLASTS** | |
| **MARKER** | **REFERENCE** |
| *S100a4* | Strutz et al. (1995) (DOI: 10.1083/jcb.130.2.393) |
| *Vim* | Strutz et al. (1995) (DOI: 10.1083/jcb.130.2.393) |
| *Col13a1* | Strutz et al. (1995) (DOI: 10.1083/jcb.130.2.393) |
| *Col14a1* | Strutz et al. (1995) (DOI: 10.1083/jcb.130.2.393) |
| *Col1a1* | Strutz et al. (1995) (DOI: 10.1083/jcb.130.2.393) |
| *Itga8* | Strutz et al. (1995) (DOI: 10.1083/jcb.130.2.393) |
| *Cxcl14* | Strutz et al. (1995) (DOI: 10.1083/jcb.130.2.393) |
| *Npnt* | Strutz et al. (1995) (DOI: 10.1083/jcb.130.2.393) |
| *Neat1* | Strutz et al. (1995) (DOI: 10.1083/jcb.130.2.393) |
| *Tcf21* | Strutz et al. (1995) (DOI: 10.1083/jcb.130.2.393) |
| *Lbh* | Strutz et al. (1995) (DOI: 10.1083/jcb.130.2.393) |
| *Nr2f2* | Strutz et al. (1995) (DOI: 10.1083/jcb.130.2.393) |
| *Tbx2* | Strutz et al. (1995) (DOI: 10.1083/jcb.130.2.393) |
| *Meox2* | Strutz et al. (1995) (DOI: 10.1083/jcb.130.2.393) |
| *Pi16* | Strutz et al. (1995) (DOI: 10.1083/jcb.130.2.393) |
| *Mmp3* | Strutz et al. (1995) (DOI: 10.1083/jcb.130.2.393) |
| *Cygb* | Strutz et al. (1995) (DOI: 10.1083/jcb.130.2.393) |
| *Rtp4* | Strutz et al. (1995) (DOI: 10.1083/jcb.130.2.393) |
| *Clec3b* | Strutz et al. (1995) (DOI: 10.1083/jcb.130.2.393) |
| *Dcn* | Strutz et al. (1995) (DOI: 10.1083/jcb.130.2.393) |
| *Prrx1* | Strutz et al. (1995) (DOI: 10.1083/jcb.130.2.393) |
| *Aebp1* | Strutz et al. (1995) (DOI: 10.1083/jcb.130.2.393) |
| *Lbh* | Strutz et al. (1995) (DOI: 10.1083/jcb.130.2.393) |
| *Meg3* | Strutz et al. (1995) (DOI: 10.1083/jcb.130.2.393) |
|  |  |
| **MARKERS_RPE** | |
| **MARKER** | **REFERENCE** |
| *Ttr* | Booij et al. (2009) (DOI:10.1186/1471-2164-10-164) |
| *Ptgds* | Booij et al. (2009) (DOI:10.1186/1471-2164-10-164) |
| *Best1* | Liao et al. (2010) (DOI: 10.1093/hmg/ddq341); Strunnikova et al. (2010) (DOI: 10.1093/hmg/ddq129) |
| *Rlbp1* | Bennis et al. (2015) (DOI: 10.1371/journal.pone.0141597) |
| *Lrat* | Liao et al. (2010) (DOI: 10.1093/hmg/ddq341) |
| *Rpe65* | Liao et al. (2010) (DOI: 10.1093/hmg/ddq341); Strunnikova et al. (2010) (DOI: 10.1093/hmg/ddq129) |
| *Rdh5* | Bennis et al. (2015) (DOI: 10.1371/journal.pone.0141597) |
| *Tyrp1* | Strunnikova et al. (2010) (DOI: 10.1093/hmg/ddq129) |
| *Krt18* | Bennis et al. (2015) (DOI: 10.1371/journal.pone.0141597) |
| *Rdh10* | Bennis et al. (2015) (DOI: 10.1371/journal.pone.0141597) |
| *Bmp4* | Bennis et al. (2015) (DOI: 10.1371/journal.pone.0141597); Strunnikova et al. (2010) (DOI: 10.1093/hmg/ddq129) |
| *Rbp1* | Bennis et al. (2015) (DOI: 10.1371/journal.pone.0141597); Strunnikova et al. (2010) (DOI: 10.1093/hmg/ddq129) |

**Supplementary Table 2. Primer sets used for RT-qPCR.** (F: forward primer; R: reverse primer)

| Target Genes | Sequences | |
| --- | --- | --- |
| Mouse *ActB* | F | GGCGGACTATGACTTAGTTG |
|  | R | AAACAATGTGCAATCAA |
| Mouse *Pkm2* | F | TGACCTGGGCATTGAGATTC |
|  | R | GAACAGCCTCCAGAGGGTAG |
| Mouse *Eno1* | F | TGCGTCCACTGGCATCTAC |
|  | R | CAGAGCAGGCGCAATAGTTTTA |
| Mouse *Pgk1* | F | ATGTCGCTTTCCAACAAGCTG |
|  | R | GCTCCATTGTCCAAGCAGAAT |
| Mouse *Aldoa* | F | CGTGTGAATCCCTGCATTGG |
|  | R | CAGCCCCTGGGTAGTTGTC |
| Mouse *Ldha* | F | TGTCTCCAGCAAAGACTACTGT |
|  | R | GACTGTACTTGACAATGTTGGGA |
| Mouse *Pgam1* | F | TCTGTGCAGAAGAGAGCAATCC |
|  | R | CTGTCAGACCGCCATAGTGT |
